# The first scene of artificial intelligence curriculum reform: longitudinal evidence on teachers' stress and multidimensional anxiety

**DOI:** 10.3389/fpsyg.2026.1835300

**Published:** 2026-06-15

**Authors:** Jiang Li

**Affiliations:** School of Education, Hanjiang Normal University, Shiyan, Hubei, China

**Keywords:** artificial intelligence curriculum, longitudinal tracking, school support, teaching anxiety, teaching stress

## Abstract

**Introduction:**

The formal incorporation of artificial intelligence courses into basic education has raised a key issue for the effective implementation of the curriculum: whether teachers can withstand the continuously rising teaching pressure in the early phase of reform and prevent such pressure from further developing into teaching anxiety. Limited attention has been paid to the persistent influence of teachers' perceived AI teaching stress on teaching anxiety and the potential buffering role of school support in this process.

**Methods:**

Drawing on two-wave longitudinal tracking data, this study analyzed 553 valid primary and secondary school teachers at T1 and 348 matched teachers at T2 to examine the persistent impact of perceived AI teaching stress on teaching anxiety, as well as the buffering effect of school support.

**Results:**

Teachers' stress and anxiety levels at T2 were both higher than those at T1, indicating an upward trend for both in the early stage of reform. Stress exerted a stable and significant positive effect on anxiety: stress at T1 significantly predicted anxiety at T1 (β ranging from 0.382 to 0.428, *p* < 0.001), and stress at T2 also significantly predicted anxiety at T2 (β ranging from 0.361 to 0.406, *p* < 0.001). Further analyses revealed clear cross-time continuity in both stress and anxiety, with stress at T1 significantly predicting stress at T2 (β ranging from 0.518 to 0.523, *p* = 0.001). Additionally, emotional support and training support significantly weakened the strength of the association between stress and anxiety.

**Discussion:**

The findings indicate that teacher anxiety in the early phase of artificial intelligence curriculum reform is not a short-term psychological fluctuation, but a sustained psychological risk. This highlights the necessity for schools to identify such anxiety early and implement systematic interventions to address it.

## 1 Introduction

Against the backdrop of global educational digital transformation, artificial intelligence curriculum reform is shifting from exploratory technology application toward the restructuring of the basic education curriculum system. Internationally, UNESCO has released an artificial intelligence competency framework for teachers, emphasizing that teachers need to develop integrated competencies in artificial intelligence ethics, basic principles, instructional application, and professional development. At the same time, countries such as Singapore, South Korea, and the United States have incorporated artificial intelligence literacy into basic education reform through artificial intelligence learning platforms, artificial intelligence digital textbooks, computational thinking, and artificial intelligence curricula, and teacher training programs.

China has also positioned artificial intelligence as an important direction in its educational digitalization strategy and issued the National Digital Education Action Plan (Ministry of Education of the People's Republic of China, 2024). Beijing, Shanghai, Hangzhou, and Guangzhou, as the first pilot cities, formally incorporated artificial intelligence into the curriculum system of basic education across all school levels from September 2025 onward. This reform requires teachers to master the basic principles of artificial intelligence, interdisciplinary instructional design methods, and relevant ethical norms. It therefore goes beyond the scope of traditional training focused on instrumental technology use. These demanding competence requirements have already generated underexplored perceived AI teaching stress and occupational anxiety among teachers. The early phase of policy implementation is a critical period for teachers' psychological adaptation and support-based intervention (Chiang et al., 2025; Yue et al., 2023). If stress and anxiety are not alleviated in a timely manner during this stage, teaching quality may decline, reform implementation may be hindered, and teacher attrition may even increase (Tikkanen et al., 2020). Addressing this issue has important practical value for improving the policy system for artificial intelligence education, enhancing teachers' occupational wellbeing, and ensuring the sustainable implementation of China's digital education strategy.

Existing studies have extensively examined teacher stress and occupational anxiety, typically drawing on Conservation of Resources Theory and the Technology Acceptance Model as major theoretical foundations. The former emphasizes that when individuals face threats to resources, actual resource loss, or resource investment that fails to yield expected returns, they are more likely to experience stress reactions and negative emotions (Hobfoll and Shirom, 2000). The latter suggests that individuals' perceived usefulness and perceived ease of use of new technologies further shape their acceptance attitudes and subsequent behavioral performance (Davis, 1989). On this basis, review studies have shown that technological change in education is often accompanied by rising levels of teacher stress and anxiety, especially when requirements for technology integration change rapidly, teacher training is insufficient, or support conditions are inadequate (Fernández-Batanero et al., 2021; Henderson and Corry, 2021).

More specifically, regarding the formation of technology-related stress, empirical studies have found that insufficient knowledge of technology integration, low computer self-efficacy, and inadequate school support significantly increase teachers' technostress. By contrast, stronger technology integration competence, higher self-efficacy, and greater organizational support help buffer stress during technology use and enhance teachers' subsequent intention to use technology (Dong et al., 2020; Joo et al., 2016; Özgür, 2020). In addition, in contexts where technology use carries a compulsory character, technological complexity, privacy concerns, pressure on professional identity, and the absence of peer and administrative support further intensify teachers' anxiety and technostress (Khlaif et al., 2023).

Although considerable progress has been made, three practical issues still require further attention. First, existing evidence has mainly focused on general educational technology integration, online teaching, or the policy context of the double reduction reform. Research on teachers' psychological adaptation in the early phase of artificial intelligence curriculum reform remains limited, particularly from a longitudinal perspective. Existing policy studies indicate that educational reform alters teachers' work demands and wellbeing and may trigger occupational anxiety, stress, and burnout. High-pressure accountability policies may further increase teacher stress and strengthen intentions to leave the profession (Ao et al., 2023; Li and Yao, 2022; Ryan et al., 2017; Von der Embse et al., 2016; Yue et al., 2023). Without continuous identification of teachers' psychological trajectories in the early phase of reform, policymakers cannot match support measures in a timely way or accurately judge how stress and anxiety evolve. Second, there is still a lack of systematic empirical evidence on whether policy support and school support can serve as key external resources that buffer the pressure of artificial intelligence curriculum reform and interrupt its transmission into occupational anxiety. Third, given that teachers at different school levels face different curricular tasks, teaching contexts, and adaptation requirements, the generative logic of artificial intelligence curriculum reform pressure and its mechanism of influence on occupational anxiety may also differ. These differences across school levels warrant further examination (Chou and Chou, 2021).

To address these practical issues, this study adopted a two-wave longitudinal survey design, with T1 conducted in October 2025 and T2 conducted in December 2025. It further combined multi-group comparisons across school levels with a mixed-methods approach integrating quantitative and qualitative evidence. The longitudinal design was used to capture the dynamic changes in teachers' psychological states during the early phase of AI curriculum reform (Farrell, 1994).

Comparisons across school levels were used to identify differences in the sources and patterns of perceived AI teaching stress, the manifestations of AI teaching anxiety, and teachers' needs for school support. Meanwhile, this study combined longitudinal structural equation modeling with subsequent thematic analysis of interviews to examine, from a temporal perspective, the association pattern between teachers' perceived AI teaching stress and AI teaching anxiety, and to further identify the buffering role and boundary conditions of school support in the early stage of reform. The theoretical value of this study lies in introducing Conservation of Resources Theory, Social Support Theory, and Ecological Systems Theory into the context of artificial intelligence curriculum reform, thereby revealing the staged characteristics of teachers' psychological adaptation. Its methodological value lies in constructing a longitudinal mixed-methods framework for studying reform-related teaching stress in educational technology contexts. Its practical value lies in providing empirical evidence from frontline teachers for schools to build more targeted support systems and for policymakers to optimize artificial intelligence education policy.

## 2 Literature review

### 2.1 Theoretical framework

**Figure 1:**
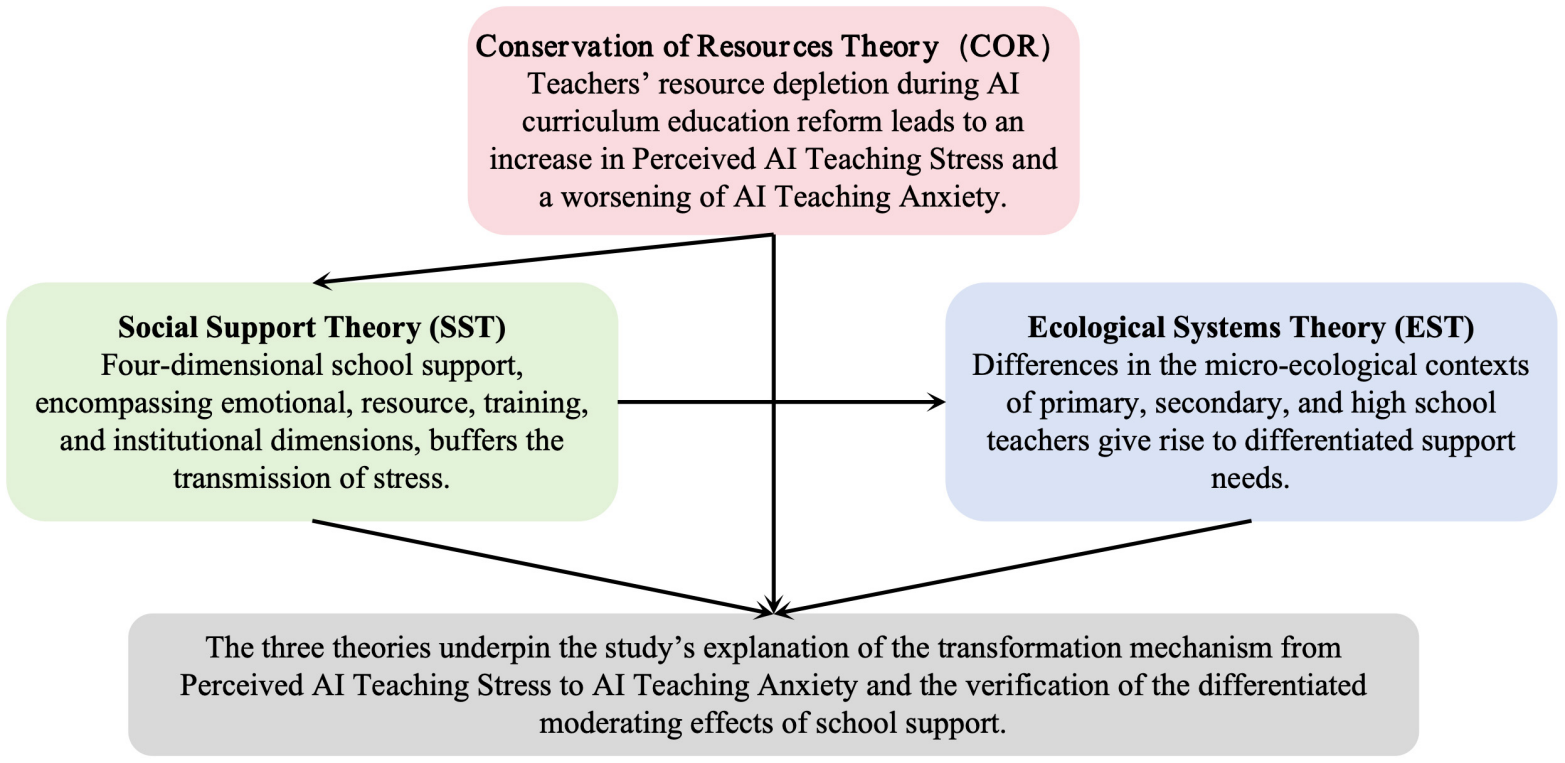
Theoretical framework of the study.

The theoretical framework of this study is grounded in Conservation of Resources Theory, Social Support Theory, and Ecological Systems Theory, as shown in Figure 1.

Conservation of Resources Theory holds that resource depletion is closely associated with stress and negative emotions, and that continued resource loss may increase psychological risk (Hobfoll, 1989). In the early phase of artificial intelligence curriculum reform, teachers must invest additional time, energy, and cognitive resources beyond their existing tasks in order to redesign curricula, respond to classroom uncertainty, and manage new instructional demands. Such sustained investment may be associated with resource depletion and insufficient recovery, which may be reflected in heightened perceived AI teaching stress and more severe AI teaching anxiety.

Social Support Theory emphasizes that organizational support can enhance wellbeing through a main effect and can also serve a buffering role in high-stress contexts by reducing the strength of the relationship between stress and psychological or physical strain (Cohen and Wills, 1985). House (1983) classified support into emotional, instrumental, and informational forms, while (Eisenberger et al. 1986) and Rhoades and Eisenberger (2002) further extended this framework in organizational settings to include institutional support. This provides the basis for the four-dimensional school support framework used in the present study, covering emotional support, resource support, training support, and institutional support. In this study, these four forms of school support are conceptualized as key external resources that may weaken the positive association between perceived AI teaching stress and AI teaching anxiety.

Ecological Systems Theory proposes that individual development and adaptation do not occur in a vacuum, but are embedded in concrete microecological environments. For teachers, curricular goals, student developmental characteristics, classroom organization, evaluation requirements, and resource allocation differ across school levels. Consequently, the sources of stress teachers perceive during artificial intelligence curriculum reform, their expressions of anxiety, and their support needs may also differ. Existing multigroup studies have shown that teachers at different instructional levels may not show identical patterns of technostress and support effects, which provides a practical basis for examining school-level differences in the present study (Bronfenbrenner, 1979; Chou and Chou, 2021).

Taken together, Conservation of Resources Theory provides a lens for understanding why perceived AI teaching stress may be associated with AI teaching anxiety, Social Support Theory clarifies the potential buffering role of school support, and Ecological Systems Theory defines the contextual boundaries in which teachers at different school levels are situated. Together, these three theories support the present study in examining teachers' stress and anxiety during the early phase of artificial intelligence curriculum reform and in testing the differentiated moderating roles of school support.

### 2.2 Conceptual model and hypotheses

**Figure 2:**
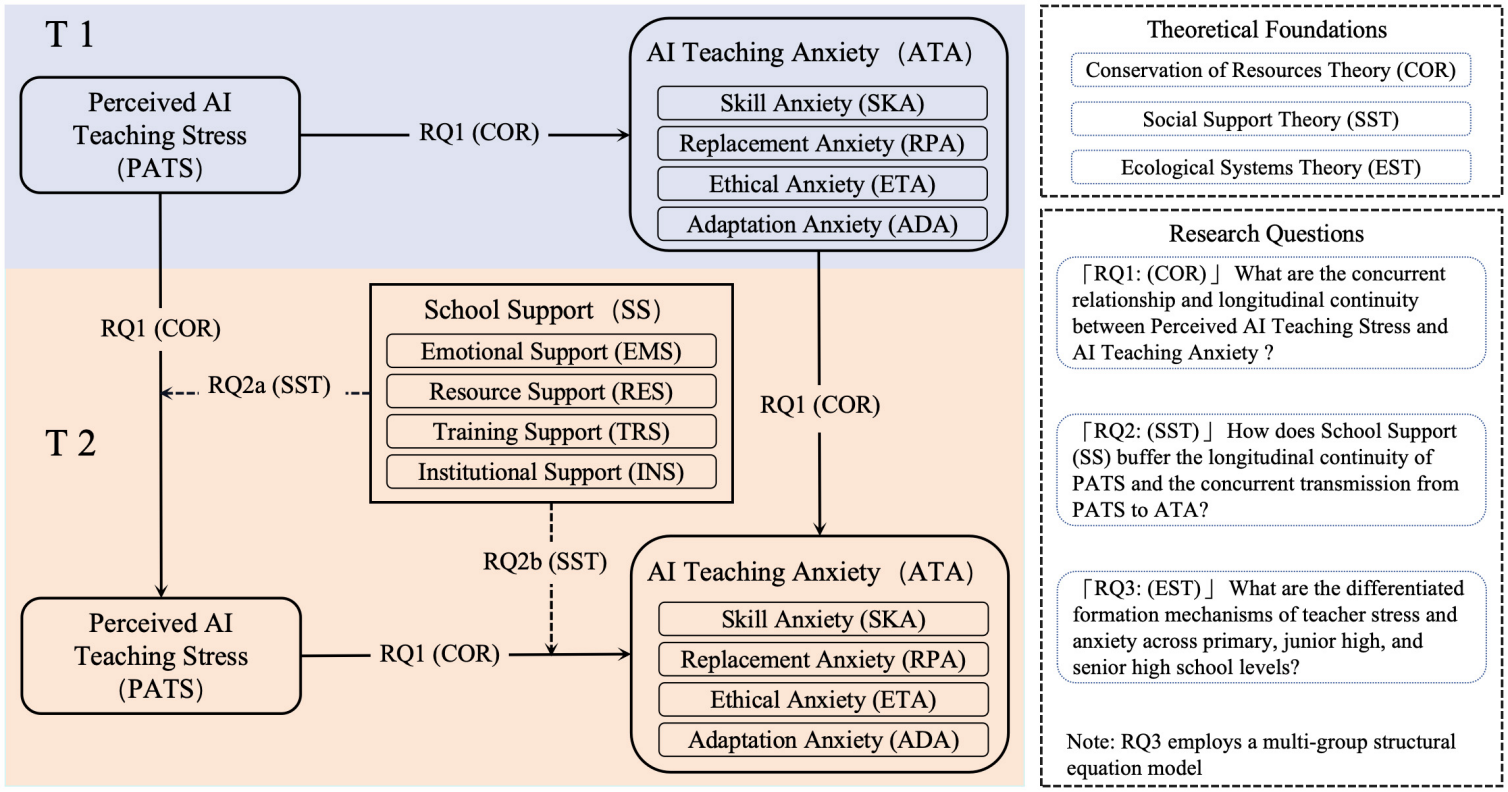
Conceptual framework of the study.

The conceptual framework of this study is built on Conservation of Resources Theory, Social Support Theory, and Ecological Systems Theory. By integrating a two-time-point design, the relations among the core variables, and the contextual boundaries associated with school level, the framework systematically addresses three major research questions. The overall logic is shown in Figure 2.

#### 2.2.1 Perceived AI teaching stress and AI teaching anxiety

Perceived AI teaching stress refers to teachers' overall perception of stress in the early phase of artificial intelligence curriculum reform, arising from heightened curricular demands, insufficient preparedness, role repositioning, and increased instructional uncertainty. Teacher stress is commonly understood as a mismatch between work demands and available resources, and educational reform often intensifies such a mismatch (Kyriacou and Chris, 2001). Recent reform research has likewise shown that policy change can alter teachers' work characteristics and wellbeing, thereby increasing stress and anxiety (Ao et al., 2023).

It is important to clarify that perceived AI teaching stress is not equivalent to technostress in the narrow sense. Reviews of educational technology have indeed shown that technological change can heighten teacher stress and anxiety (Fernández-Batanero et al., 2021; Henderson and Corry, 2021). However, artificial intelligence curriculum reform is more complex than ordinary technology adoption. It involves not only tool use, but also understanding artificial intelligence concepts, designing curriculum, making ethical judgments, and complying with data privacy and safety regulations (Akgun and Greenhow, 2022; European Commission, 2022). Reviews of K-12 artificial intelligence education have likewise pointed out that artificial intelligence curriculum design must simultaneously address technical knowledge and ethical issues, which places teachers under a more complex set of adaptive instructional demands (Li, 2022). Accordingly, this study defines perceived AI teaching stress as a form of comprehensive instructional stress situated in the reform context, of which technology-related burden is only one component.

AI teaching anxiety refers to the multidimensional emotional response teachers experience in the context of artificial intelligence curriculum reform when confronted with insufficient competence, loss of classroom control, ethical risk, curricular mismatch, and occupational uncertainty. Anxiety is generally defined as a state of tension, worry, and vigilance in response to potential threats and uncertain outcomes (Zung, 1971). In artificial intelligence education, scholars have pointed out that teachers must simultaneously address ethical challenges, risks associated with data use, and issues of educational fit after artificial intelligence enters the classroom (Akgun and Greenhow, 2022). Research on artificial intelligence ethics in K-12 contexts also indicates that policy related to children and school education is still developing rapidly, and that fairness, privacy, safety, and responsibility boundaries are all core issues teachers must confront (Adams et al., 2023). It is therefore well grounded to divide AI teaching anxiety into skill anxiety, replacement anxiety, ethical anxiety, and adaptation anxiety.

From a mechanistic perspective, Conservation of Resources Theory suggests that when external demands exceed an individual's existing resource capacity, the resource gap first manifests as stress and then gives rise to negative emotions such as anxiety (Hobfoll, 1989). Longitudinal studies on teacher stress further show that teachers' stress responses vary over time and are influenced by school support and adaptation conditions (Harmsen et al., 2019). A semester-long tracking study also found that teacher wellbeing fluctuates significantly within a school term, indicating that teachers' psychological states are dynamic rather than static (Collie and Martin, 2023). On this basis, the present study needs to test both the synchronous relation between perceived AI teaching stress and AI teaching anxiety and their longitudinal continuity.

Accordingly, this study proposes research question 1: in the early phase of artificial intelligence curriculum reform, what are the synchronous relations and longitudinal continuities between perceived AI teaching stress and AI teaching anxiety? The following hypotheses are proposed:

H1: during the early phase of artificial intelligence curriculum reform, teachers' perceived AI teaching stress and AI teaching anxiety significantly increase from T1 to T2.

H2: teachers' perceived AI teaching stress is significantly and positively associated with AI teaching anxiety at both T1 and T2, and both stress and anxiety show cross-time continuity.

#### 2.2.2 School support

School support refers to teachers' overall perception of the emotional care, material resources, professional training, and institutional safeguards provided by schools during artificial intelligence curriculum reform. Social Support Theory suggests that support can directly promote wellbeing and can also exert a buffering effect when stress is high (Cohen and Wills, 1985). In organizational contexts, individuals who perceive higher levels of organizational support are generally more likely to develop a sense of security, belonging, and positive psychological adjustment (Eisenberger et al., 1986; Rhoades and Eisenberger, 2002). Therefore, in the early phase of artificial intelligence curriculum reform, school support is not merely a general external condition, but also a potentially important protective resource that can mitigate the accumulation of stress and the transmission of anxiety.

The three support dimensions proposed by House (1983) were extended by Eisenberger et al. (1986) in educational contexts to include institutional support, forming the four-dimensional framework adopted in this study. The empirical value of each support dimension has been demonstrated. Emotional support can strengthen teachers' psychological safety and alleviate concerns about occupational replacement (Eisenberger et al., 1986; Thoits, 2011), although its short-term buffering effect remains somewhat uncertain (Berkovich and Eyal, 2020). Resource support is crucial for alleviating teacher stress in the early phase of technological change (Khlaif et al., 2023), and its availability directly affects teaching confidence (Morska et al., 2022). Training support can help fill teachers' competence gaps in artificial intelligence (Barajas Motta et al., 2025), although current supply does not fully match demand (Cinganotto and Montanucci, 2025). Institutional support can reduce role ambiguity, but poorly designed institutional arrangements may also amplify stress (Haq, 2025). The buffering hypothesis of social support suggests that high-quality support weakens the transmission from stress to anxiety (Cohen and Wills, 1985; Rhoades and Eisenberger, 2002), and that different forms of support may buffer stress differently, with resource support being more salient in the early phase of reform and institutional support increasing in importance over time (Li, 2022).

As AI curriculum reform continues to advance, the stress formed by teachers in the early stage does not necessarily dissipate naturally; instead, it may persist and accumulate in subsequent stages (Harmsen et al., 2019). The significance of school support in this process lies not only in alleviating teachers' current anxiety experiences, but also in weakening the continued extension of earlier stress into later stress. In other words, when teachers continuously perceive emotional support, resource support, training support, and institutional support from their schools during the reform process, a previously high level of stress is less likely to be further reinforced at a later stage. At the same time, under similar levels of stress, teachers who receive more school support are also less likely to show a high level of AI teaching anxiety. On this basis, this study examines the differentiated buffering effects of school support from two aspects: first, the mitigating effect of school support on the longitudinal continuity of perceived AI teaching stress; and second, the mitigating effect of school support on the relationship between perceived AI teaching stress and AI teaching anxiety at T2.

Accordingly, this study proposes Research Question 2: how does school support mitigate the longitudinal continuity of teachers' perceived AI teaching stress and the relationship between perceived AI teaching stress and AI teaching anxiety at T2? The following hypotheses are proposed:

H3: school support significantly weakens the longitudinal continuity of teachers' perceived AI teaching stress.

H4: school support significantly weakens the positive relationship between perceived AI teaching stress and AI teaching anxiety at T2.

#### 2.2.3 Differences across school levels

Differences across school levels refer to variations in perceived stress, sources of anxiety, and support needs during artificial intelligence curriculum reform among primary school, junior secondary school, and senior secondary school teachers, arising from differences in teaching goals, students' cognitive characteristics, and other microecological conditions. Ecological Systems Theory suggests that individuals' needs and behavioral responses are directly shaped by the microecological environment, and that effective support must match environmental characteristics (Bronfenbrenner, 1977). In the context of artificial intelligence curriculum reform, primary school instruction emphasizes cognitive initiation and engaging interaction, creating greater demands for accessible resources and interactive equipment. Secondary education, by contrast, is more strongly oriented toward knowledge construction and examination requirements, and therefore places greater emphasis on disciplinary depth and ethical compliance in artificial intelligence teaching (Li, 2022). Empirical research has shown that primary school teachers rely more heavily on material resource support, whereas secondary school teachers place greater value on institutional safeguards. These differences stem from the fundamentally different goals of teaching across school levels (Chou and Chou, 2021). On this basis, the moderating effect of school support is expected to vary by school level. Accordingly, research question 3 is proposed: what are the differentiated mechanisms through which stress and anxiety emerge among primary school, junior secondary school, and senior secondary school teachers? The following hypothesis is proposed:

H5: the buffering effect of school support on the relationship between stress and anxiety shows differentiated patterns across teachers at different school levels.

## 3 Method

### 3.1 Research design

This study adopted a mixed-methods design combining a two-wave longitudinal questionnaire survey with qualitative interviews. The T1 survey was conducted from October 1 to 15, 2025, corresponding to the initial stage after artificial intelligence curriculum reform entered school teaching practice. The T2 survey was conducted from December 1 to 15, 2025, corresponding to the adaptation stage after teachers had experienced a period of curriculum implementation. Both surveys measured teachers' perceived AI teaching stress and AI teaching anxiety in order to identify changes over time, synchronous associations, and cross-time continuity in the core psychological variables. School support was measured at T2, mainly to examine the level of support teachers actually perceived during the implementation of the reform and its buffering effect.

**Figure 3:**
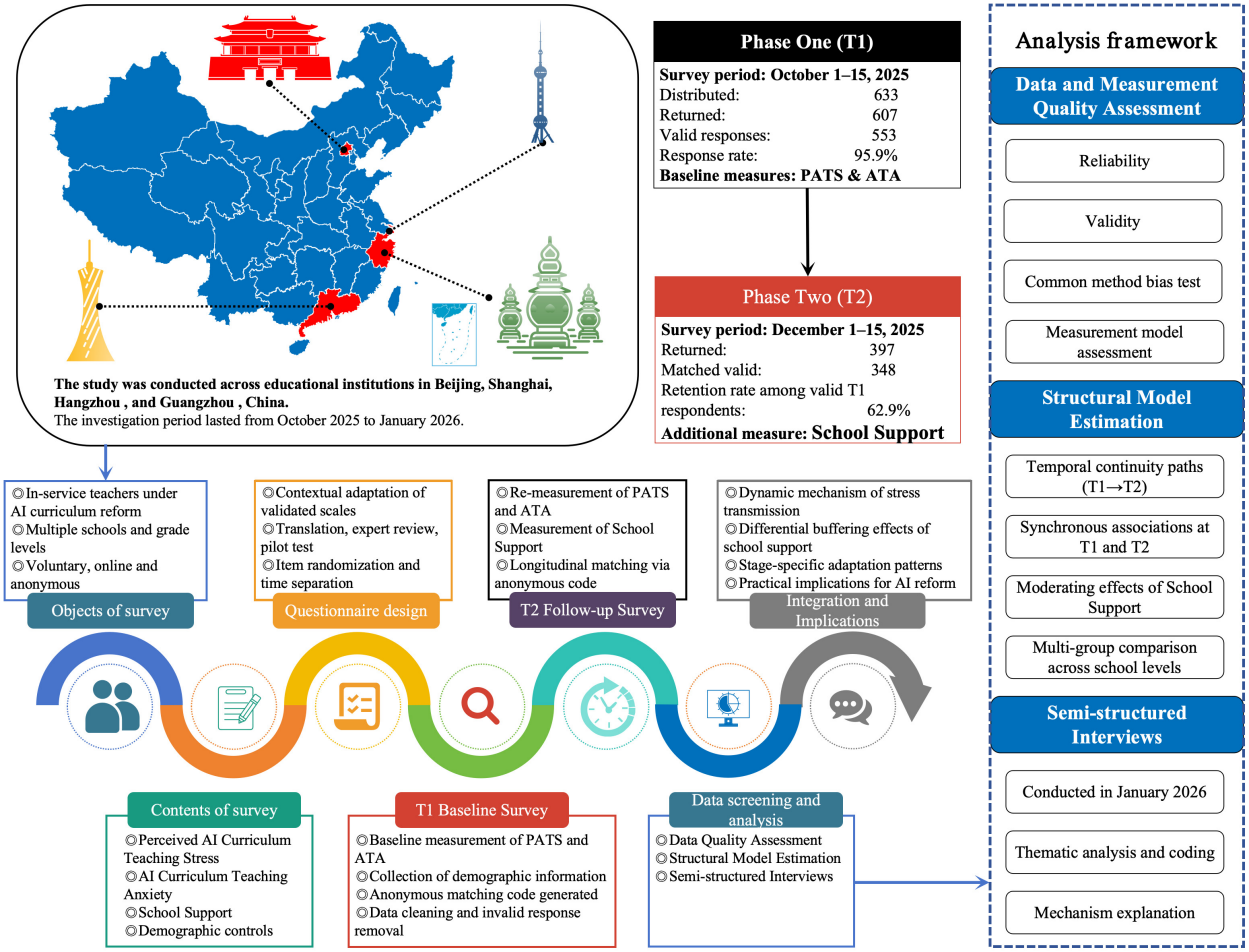
Research process and analytical framework.

The quantitative part of the study consisted of four main analytical steps. First, changes in perceived AI teaching stress and AI teaching anxiety from T1 to T2 were compared. Second, the associations between stress and anxiety at the same time point and the cross-time continuity of the two variables were examined. Third, the study tested whether school support weakened the longitudinal continuity of stress and whether it weakened the positive relationship between stress and anxiety at T2. Fourth, multi-group analysis was used to compare structural path differences among primary school, junior secondary school, and senior secondary school teachers. The qualitative interviews were mainly used to supplement and explain the quantitative results and to present teachers' sources of stress, anxiety experiences, and support needs during artificial intelligence curriculum reform. The research procedure is shown in Figure 3.

#### 3.1.1 Research context, participants, and sampling strategy

The research context comprised primary and secondary schools in Beijing, Shanghai, Guangzhou, and Hangzhou, where artificial intelligence curriculum reform had already been advanced or piloted. A stratified cluster sampling strategy was adopted. Cities were first used as strata, and within each city schools were further stratified by school level and selected as cluster units. Two primary schools, two junior secondary schools, and two senior secondary schools were selected in each city, yielding a total of eight primary schools, eight junior secondary schools, and eight senior secondary schools. Questionnaires were then distributed by teaching-research group or grade-level group within the selected schools. The inclusion criterion was being an in-service teacher engaged in teaching, teaching research, or management related to artificial intelligence curricula.

Sample size planning was determined according to the requirements of longitudinal structural equation modeling, latent variable moderation testing, and multi-group comparisons across school levels. This study involved three core variables: perceived AI teaching stress, measured at both T1 and T2 with 10 items; AI teaching anxiety, measured at both T1 and T2 with 21 items; and school support, measured at T2 with 26 items. Together, these instruments contained 57 observed items. Sample size estimation referred to the statistical power setting of α = 0.05, 1–β = 0.90, and a medium effect size of f^2^ = 0.15 (Cohen, 2013), and also considered the requirements of structural equation modeling for model complexity, number of latent variables, number of observed indicators, and sample size for group comparisons (Kline, 2023; Wolf et al., 2013). Considering possible attrition across the two waves, this study appropriately expanded the T1 sample size and set the target number of valid matched T2 cases at no fewer than 300, with each of the three school-level subsamples expected to approach approximately 100 cases. This was intended to meet the basic requirements for longitudinal path analysis, moderation testing, and multi-group comparison.

#### 3.1.2 Measurement instruments

All scales used in this study were adapted from internationally established instruments with strong evidence of reliability and validity. The adaptations were contextualized for artificial intelligence curriculum reform without changing the core psychological meaning of the constructs. Scale translation and revision followed the procedures of independent forward translation, synthesis, backward translation, expert committee review, and pilot testing with a small sample in order to ensure semantic and conceptual equivalence (Beaton et al., 2000). In particular, the back-translation procedure followed the classical principles used in cross-cultural research (Beaton et al., 2000). To reduce the risk of common method bias, the questionnaire was designed with procedural remedies such as temporal separation and random ordering of items, and corresponding statistical checks were later applied.

Perceived AI teaching stress was adapted from the perceived stress scale developed by Cohen and colleagues (Cohen and Williamson, 1988; Cohen et al., 1983). The original scale is mainly used to measure individuals' subjective perceptions of unpredictability, uncontrollability, and overload in recent life situations. Existing psychometric studies have shown that the 10-item version of the Perceived Stress Scale has relatively stable reliability and structural validity (Taylor, 2015). This study retained the core meaning of perceived stress and revised the general life-context wording into situations related to artificial intelligence curriculum teaching reform, in order to reflect teachers' stress experiences during reform implementation.

AI teaching anxiety was adapted from the Artificial Intelligence Anxiety Scale developed by Wang (Wang and Wang, 2022). The original scale measures individuals' anxiety when facing artificial intelligence learning, application, and sociotechnical risks. It includes dimensions such as AI learning anxiety, job replacement anxiety, sociotechnical blind spot anxiety, and AI configuration anxiety. The scale has been used in subsequent studies on artificial intelligence anxiety and has shown good reliability and structural validity (Terzi, 2020; Wang and Wang, 2022). In this study, based on the context of teachers' artificial intelligence curriculum teaching reform, the general wording related to AI learning and application in the original scale was revised into expressions concerning AI curriculum knowledge mastery, AI teaching tool use, classroom integration, ethical risks, and changes in teachers' professional roles. This resulted in four dimensions: skill anxiety, replacement anxiety, ethical anxiety, and adaptation anxiety.

School support included four dimensions: emotional support, resource support, training support, and institutional support. Emotional support and resource support were adapted from the perceived support subscale of the Berlin Social Support Scales (Schulz and Schwarzer, 2003). These dimensions were used to measure teachers' perceived emotional care, colleague support, resource provision, and practical assistance during artificial intelligence curriculum reform. Emotional support included four items, mainly reflecting the role of school leaders, colleagues, and teaching-research teams in emotional care, listening and understanding, and psychological support. Training support and institutional support were adapted from the Implementation Climate Scale developed by Ehrhart et al. (2014). The original scale was designed to measure the supportive implementation climate formed within organizations during the implementation of evidence-based practices, covering educational support, recognition, rewards, goal emphasis, staff selection, and openness. In this study, the educational support dimension was adapted into training support for AI curriculum teaching, while the other dimensions related to organizational implementation climate were adapted into institutional support in the context of artificial intelligence curriculum reform. Training support included three items and mainly reflected schools' support in AI curriculum teaching training, classroom integration guidance, and ethical norms training.

To ensure the measurement basis for subsequent structural path analysis, this study further examined item loadings and discriminant validity for each latent variable. The standardized factor loadings were generally within an acceptable range. Discriminant validity was assessed using the heterotrait-monotrait ratio, and the results showed that the core latent variables had generally acceptable discriminant validity. The full item loadings, heterotrait-monotrait ratio results, and supplementary measurement model results are presented in Supplementary material 1, Appendix 3, Tables S3a–S6.

#### 3.1.3 Phase 1 (T1)

The T1 survey was conducted from October 1 to 15, 2025. The research team distributed the online questionnaire link to relevant in-service teachers through school teaching-research departments and curriculum management channels. The first page of the questionnaire provided a study description and informed consent information. Participation was completely voluntary, and participants could withdraw at any time. T1 mainly collected teachers' demographic information and measured their perceived AI teaching stress and AI teaching anxiety. To enable cross-wave matching, an eight-character alphanumeric random code was generated by the survey platform after T1 submission, and participants were asked to save this code for the follow-up survey. At T1, 633 questionnaires were distributed and 607 were returned. After data quality control, 553 valid responses were retained.

Data quality control procedures for T1 were specified in advance and implemented uniformly. First, two instructional attention-check items were included, and any respondent who failed either item was excluded. Second, a response-time threshold was set based on the pilot distribution, and any total completion time below 180 s was treated as low-quality and excluded. Third, single-option responding and long-string response patterns were screened. Cases were removed if the same response option accounted for 90% or more of the core scale items or if long strings of identical responses appeared across scales. Fourth, cases with more than 10% missing data on key variables were not included in the analysis.

#### 3.1.4 Phase 2 (T2)

The T2 survey was conducted from December 1 to 15, 2025 and was sent only to valid T1 participants. The first item of the T2 questionnaire asked participants to enter the matching random code obtained at T1 in order to link the two waves of data. Cases that could not be matched were not included in the longitudinal analysis. T2 repeated the measurement of perceived AI teaching stress and AI teaching anxiety, and additionally measured school support, including emotional support, resource support, training support, and institutional support. To improve sample retention, the research team sent up to two reminders without exerting pressure and provided equivalent non-cash incentives. A total of 397 questionnaires were returned at T2. After successful matching and data quality control, 348 valid responses were retained, corresponding to a retention rate of 62.9% relative to the valid T1 sample.

The T2 data quality control rules were the same as those used at T1, while the response-time threshold was set at 180 s in view of the larger number of items. Cases showing obvious contradictions across waves were reviewed and excluded. To assess the risk of selective attrition, the retained group and the attrition group were compared in terms of core T1 variables and demographic characteristics. If necessary, inverse probability weights were introduced into the model as a sensitivity analysis. The final analytic sample used in the structural models is reported in the Results section.

#### 3.1.5 Qualitative interviews

Qualitative interviews were conducted in January 2026, mainly to explain the quantitative findings and to present how school support operated during artificial intelligence curriculum reform. To reduce concerns about being evaluated and the risk of socially desirable responses, schools only assisted in forwarding the questionnaire and interview recruitment information. They did not participate in sample screening, interviewee selection, or data analysis, nor did they have access to teachers' individual questionnaires, random codes, interview lists, or interview content. Teachers who were willing to participate in the interviews left their contact information through an independent link. Contact information was stored separately from the questionnaire data and was not included in the quantitative analysis dataset.

Interview participants were selected from teachers who had completed the T2 questionnaire and passed the data quality control procedures, while taking into account differences in school support levels, anxiety levels, school level, and school type. A total of ten interviewees were selected. Before the interviews, the researchers again explained the research purpose, confidentiality principles, and participants' right to withdraw, and clearly stated that interview participation was unrelated to school evaluation, performance appraisal, or curriculum reform assessment. The interviews followed a semi-structured protocol and focused on sources of reform-related stress, anxiety-triggering situations, the accessibility of school support, and the process through which school support functioned. With the oral consent of the interviewees, the interviews were audio-recorded, transcribed, and de-identified. Thematic analysis was then used for coding and theme generation (Braun and Clarke, 2006). The qualitative results were used only to supplement and explain the structural relationships shown in the quantitative model and were not used as a basis for individual evaluation.

### 3.2 Data processing and statistical analysis

Data cleaning and missingness assessment were conducted first. Missing data patterns and Little's test were used to determine whether the missingness approximated missing completely at random (Little, 1988). When the proportion of missing data was acceptable, multiple imputation was used with five imputations in order to reduce sample loss and obtain robust estimates (Enders, 2022; Rubin, 1987). Common method bias was then examined. In addition to the procedural remedy of temporal separation, Harman's single-factor test was applied, and a method factor was included in the structural model as a sensitivity analysis (Podsakoff et al., 2003). Structural equation modeling served as the main analytical framework. At the measurement level, confirmatory factor analyses were conducted for perceived AI teaching stress, the four dimensions of AI teaching anxiety, and the four dimensions of school support. Internal consistency, composite reliability, average variance extracted, and the heterotrait-monotrait ratio were reported, and model fit was evaluated using commonly accepted fit indices (Hu and Bentler, 1999). To ensure the interpretability of comparisons across time and across groups, repeated constructs at T1 and T2 were tested sequentially for configural invariance, metric invariance, and scalar invariance (Vandenberg and Lance, 2000), using fit change thresholds as the decision criteria (Chen, 2007). At the structural level, the temporal stability paths for stress and anxiety were estimated, and after controlling for baseline anxiety at T1 and other control variables, the synchronous relation between stress and anxiety at T2 was tested. Moderating effects were examined primarily through latent variable interactions. Specifically, the study tested whether each dimension of school support moderated the path from perceived AI teaching stress at T1 to perceived AI teaching stress at T2 and the path from perceived AI teaching stress at T2 to AI teaching anxiety at T2. Simple slopes and interval estimates were reported, and latent moderated structural equations could be used to estimate these interactions (Chen, 2007). Given the potential within-school dependence induced by cluster sampling, cluster-robust standard errors or complex survey corrections were applied to avoid underestimating standard errors.

### 3.3 Ethics and compliance

Before implementation, this study received ethical approval from the School Ethics Committee of the School of Education, Hanjiang Normal University, Shiyan, China (September 20, 2025; Ref: HJNU/SRC/SFL/30). The study was conducted in accordance with the approved protocol and followed the international ethical standards of the Declaration of Helsinki and its subsequent amendments. All participants provided informed consent voluntarily and had the right to withdraw at any time. No directly identifiable personal information was collected, and all data were used solely for academic research and kept confidential. Any unforeseen issues arising during the study were to be reported promptly to the Ethics Committee.

## 4 Results

### 4.1 Sample characteristics and preliminary data checks

Table 1 presents the final matched sample included in the longitudinal model analysis, consisting of 348 primary and secondary school teachers. The sample covered three school levels: 109 primary school teachers, 123 junior secondary school teachers, and 116 senior secondary school teachers. Female teachers accounted for 56.3% of the sample, while male teachers accounted for 43.7%. The age distribution was mainly concentrated between 35 and 50 years, accounting for 58.0% of the total sample. Teachers with more than 10 years of teaching experience accounted for 55.7%, indicating that the sample mainly consisted of mid-career teachers and teachers with relatively substantial teaching experience. The subjects taught included Chinese, mathematics, English, science, information technology, integrated courses, and other subjects, indicating a certain degree of heterogeneity in the sample.

**Table 1:** Demographic characteristics.

Category	Group	Frequency	Percentage
Gender	Male	152	43.7
	Female	196	56.3
Age group	Below 35 years	76	21.8
	35-50 years	202	58
	Above 50 years	70	20.1
Years of teaching	Below 5 years	58	16.7
	5-10 years	96	27.6
	More than 10 years	194	55.7
School level taught	Primary school	109	31.3
	Junior secondary school	123	35.3
	Senior secondary school	116	33.3
Teaching subject	Chinese	63	18.1
	Mathematics	85	24.4
	English	59	17
	Science	57	16.4
	Information technology	56	16.1
	Integrated curriculum	14	4
	Other	14	4
Household registration	Local	132	37.9
	Urban migrant	140	40.2
	County migrant	76	21.8
Self-rated literacy	Low literacy	76	21.8
	Moderate literacy	202	58
	High literacy	70	20.1

To examine whether longitudinal attrition might have caused systematic bias, this study compared the retained T2 sample with the attrition sample in terms of core T1 variables and major demographic variables. The results showed that there were no significant differences between the retained and attrition samples in T1 perceived AI teaching stress, T1 AI teaching anxiety, gender, age, teaching experience, or school-level distribution. This indicates that sample attrition did not show obvious systematic bias. The data quality checks showed that all final cases passed the attention checks, response-time screening, and regular response pattern detection, indicating that the sample was suitable for subsequent longitudinal structural equation modeling analysis.

### 4.2 Descriptive statistics and correlation analysis

Before testing the structural model, this study first conducted descriptive statistics and correlation analyses for the core variables. Detailed descriptive results are presented in Supplementary material 1, Appendix 2, Table S2. The results showed that teachers had already reported moderately high levels of perceived AI teaching stress and AI teaching anxiety at the first wave. The mean score of T1 perceived AI teaching stress was 3.685, with a standard deviation of 1.067. The mean score of overall T1 AI teaching anxiety was 3.596, with a standard deviation of 1.012. At T2, the mean scores of the core variables increased overall. The mean score of T2 perceived AI teaching stress was 3.879, and the mean score of overall T2 AI teaching anxiety was 3.924.

**Figure 4:**
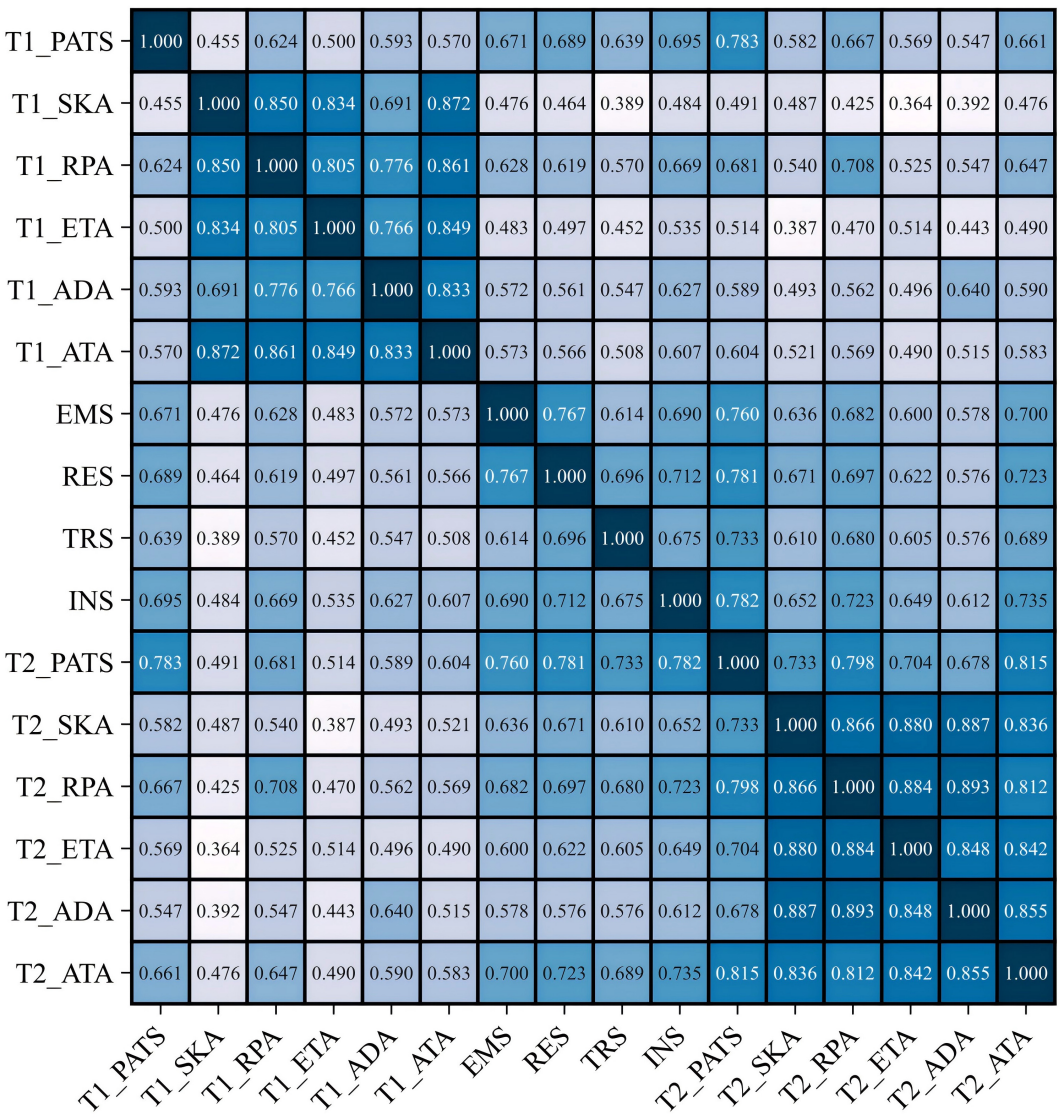
Correlation matrix of study variables. T1, Time 1; T2, Time 2; PATS, Perceived AI Teaching Stress; ATA, AI Teaching Anxiety; SKA, Skill Anxiety; RPA, Replacement Anxiety; ETA, Ethical Anxiety; ADA, Adaptation Anxiety; EMS, Emotional Support; RES, Resource Support; TRS, Training Support; INS, Institutional Support. Values are Pearson correlation coefficients.

As shown in Figure 4, the correlation analysis indicated that perceived AI teaching stress was significantly and positively correlated with AI teaching anxiety and its four dimensions at both T1 and T2. The same constructs were also significantly and positively correlated between T1 and T2, preliminarily suggesting a certain degree of temporal stability in both stress and anxiety. These results provided a basis for the subsequent tests of time changes, synchronous associations, and cross-time continuity.

### 4.3 Changes in perceived AI teaching stress and AI teaching anxiety from T1 to T2

This section tests H1, which states that perceived AI teaching stress and AI teaching anxiety significantly increased from the first wave to the second wave during the early phase of artificial intelligence curriculum reform. The results of the repeated-measures analysis are shown in Table 2.

**Table 2:** Repeated measures.

Variable	T1 (M, SD)	T2 (M, SD)	*F*	Difference	*P*	ηp^2^
PATS	3.685 (1.067)	3.879 (0.954)	28.787	T1 < T2	0.001	0.077
SKA	3.517 (1.158)	3.910 (0.932)	46.330	T1 < T2	0.001	0.118
RPA	3.653 (0.997)	3.950 (0.956)	54.950	T1 < T2	0.001	0.137
ETA	3.591 (1.107)	3.920 (0.969)	35.645	T1 < T2	0.001	0.093
ADA	3.701 (1.088)	3.918 (0.959)	21.210	T1 < T2	0.001	0.058
ATA	3.596 (1.012)	3.924 (0.861)	50.018	T1 < T2	0.001	0.126

PATS, Perceived AI teaching stress; ATA, AI teaching anxiety; SKA, Skill anxiety; RPA, Replacement anxiety; ETA, Ethical anxiety; ADA, Adaptation anxiety.

The perception of teaching pressure in AI courses increased from 3.685 at T1 to 3.879 at T2, *F* = 28.787, *p* = 0.001, ηp^2^ = 0.077. The overall teaching anxiety in AI courses increased from 3.596 at T1 to 3.924 at T2, *F* = 50.018, *p* = 0.001, ηp^2^ = 0.126. The four anxiety dimensions also showed a consistent upward trend. Skill anxiety increased from 3.517 to 3.910, *F* = 46.330, *p* = 0.001, ηp^2^ = 0.118; replacement anxiety increased from 3.653 to 3.950, *F* = 54.950, *p* = 0.001, ηp^2^ = 0.137; ethical anxiety increased from 3.591 to 3.920, *F* = 35.645, *p* = 0.001, ηp^2^ = 0.093; adaptation anxiety increased from 3.701 to 3.918, *F* = 21.210, *p* = 0.001, ηp^2^ = 0.058.

From the effect size perspective, the time effect of substitution anxiety was the most significant, indicating that teachers' concerns about AI weakening the professional value of teachers and replacing some teaching functions grew rapidly in the early stage of the reform. The above results show that the pressure and anxiety of teachers during the early stage of the AI course reform both showed a significant upward trend, and H1 was supported.

### 4.4 Preliminary checks

Before testing the core paths of the structural equation model, this study first examined the reliability and validity of the measurement instruments and assessed model fit, in order to ensure the scientific rigor and reliability of the subsequent path analysis. The core results are summarized below, and the detailed data and tables are provided in Supplementary material 1, Appendix 3, Tables S3a–S6. under the detailed results of measurement assessment and model fit for the structural equation model.

#### 4.4.1 Reliability of the measurement instruments

All research scales showed satisfactory to high internal consistency and acceptable convergent validity. Cronbach's α coefficients ranged from 0.818 to 0.962, and composite reliability values were all above 0.892, exceeding the commonly recommended threshold of 0.70 for internal consistency reliability (Hair and Alamer, 2022). Although the highest reliability coefficients suggest that potential item redundancy should not be completely ruled out, the overall results indicate that the measurement instruments had stable internal consistency. The average variance extracted values ranged from 0.512 to 0.733, all exceeding the recommended minimum value of 0.50, indicating that each latent variable explained an acceptable proportion of variance in its observed indicators and thus supported convergent validity (Hair and Alamer, 2022). Detailed results are presented in Supplementary material 1, Appendix 3, Tables S3a-3e.

#### 4.4.2 Discriminant validity of the measurement instruments

Discriminant validity was examined using the heterotrait-monotrait ratio (HTMT). Following previous recommendations, HTMT values below 0.85 indicate strict discriminant validity, while values below 0.90 can be considered acceptable for conceptually related constructs (Hair and Alamer, 2022; Henseler et al., 2015). Across the five models, HTMT values ranged from 0.620 to 0.893. Most values were below 0.85, and all values were below 0.90. The highest value was found between resource support and training support (HTMT = 0.893), suggesting that these two dimensions were closely related. Overall, the results generally supported discriminant validity, although the distinction between resource support and training support should be interpreted with caution. Detailed results are presented in Supplementary material 1, Appendix 3, Tables S4a-4e.

#### 4.4.3 Convergent validity and multicollinearity

Across the measurement models, the standardized factor loadings of the observed variables on their corresponding latent variables ranged from 0.681 to 0.901. Although several loadings were below the ideal value of 0.700, all exceeded 0.60 and were therefore considered acceptable in combination with the satisfactory composite reliability and AVE results (Hair and Alamer, 2022; Hair et al., 2019). The Variance Inflation Factor (VIF) ranges from 1.547 to 8.624, all falling below the commonly used threshold of 10 (Thompson et al., 2017). Detailed results are presented in Supplementary material 1, Appendix 3, Table S5.

#### 4.4.4 Model fit assessment

The assessment of the five structural equation models showed weak-to-moderate explanatory power for the endogenous variables, with *R*^2^ values for AI teaching anxiety and its dimensions ranging from 0.338 to 0.472. Following PLS-SEM reporting guidelines, *R*^2^ was interpreted as an indicator of explanatory power rather than as a global model fit index (Hair and Alamer, 2022; Hair et al., 2019). The standardized root mean square residual (SRMR) values ranged from 0.041 to 0.050, all below the conservative threshold of 0.08, suggesting that the average discrepancy between the observed and model-implied correlations was small (Henseler et al., 2016; Hu and Bentler, 1999). The normed fit index (NFI) values ranged from 0.841 to 0.873, which were below the conventional threshold of 0.90. However, considered together with the satisfactory SRMR values and the theoretically grounded model specification, the overall model fit was regarded as acceptable but not ideal. Accordingly, the subsequent path results were interpreted cautiously as statistically supported association patterns. Detailed results are presented in Supplementary material 1, Appendix 3, Table S6.

### 4.5 Model construction and key findings

**Figure 5:**
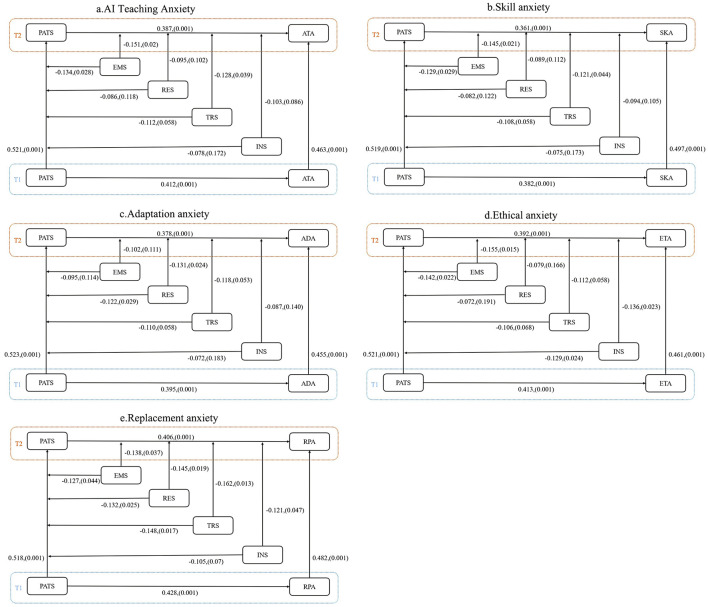
Structural equation models for AI teaching anxiety and its dimensions. (**a**) Overall AI teaching anxiety; (**b**) skill anxiety; (**c**) adaptation anxiety; (**d**) ethical anxiety; (**e**) replacement anxiety. T1, Time 1; T2, Time 2; PATS, Perceived AI Teaching Stress; ATA, AI Teaching Anxiety; SKA, Skill Anxiety; RPA, Replacement Anxiety; ETA, Ethical Anxiety; ADA, Adaptation Anxiety; EMS, Emotional Support; RES, Resource Support; TRS, Training Support; INS, Institutional Support. Values are standardized path coefficients, with *p*-values reported in parentheses.

To further examine the dynamic association mechanism between teachers' perceived stress and multidimensional anxiety in the context of artificial intelligence education, and to test the moderating effects of different dimensions of organizational support, this study constructed a series of structural equation models based on longitudinal tracking data from T1 and T2. The model paths are shown in Figure 5.

#### 4.5.1 Synchronous associations and cross-time continuity between stress and anxiety

This section tests H2, which states that teachers' perceived AI teaching stress is significantly and positively associated with AI teaching anxiety at both T1 and T2, and that both stress and anxiety show cross-time continuity. The structural equation modeling results are presented in Table 3.

**Table 3:** Structural paths for synchronous associations and cross-time continuity.

Path	β	SE	*t*	*p*
T1_PATS->T1_ATA	0.412	0.075	5.493	0.001
T2_PATS->T2_ATA	0.387	0.071	5.451	0.001
T1_PATS->T2_PATS	0.521	0.082	6.354	0.001
T1_ATA->T2_ATA	0.463	0.079	5.861	0.001

First, the synchronous path results showed that perceived AI teaching stress was significantly and positively associated with overall AI teaching anxiety at both time points. At T1, T1 perceived AI teaching stress was significantly associated with T1 overall AI teaching anxiety, β = 0.412, *T* = 5.493, *p* = 0.001. At T2, T2 perceived AI teaching stress was also significantly associated with T2 overall AI teaching anxiety, β = 0.387, *T* = 5.451, *p* = 0.001. These results indicate that, during the early phase of artificial intelligence curriculum reform, teachers who perceived higher levels of AI curriculum teaching stress also reported higher levels of synchronous AI teaching anxiety.

Second, the cross-time stability paths showed that both perceived AI teaching stress and AI teaching anxiety had significant longitudinal continuity. T1 perceived AI teaching stress significantly predicted T2 perceived AI teaching stress, β = 0.521, *T* = 6.354, *p* = 0.001, indicating that stress experiences during the early phase of reform tended to persist into the subsequent stage. T1 overall AI teaching anxiety significantly predicted T2 overall AI teaching anxiety, β = 0.463, *T* = 5.861, *p* = 0.001, indicating that teachers' AI teaching anxiety also showed cross-time stability.

Further dimension-specific model results are presented in Table 4. T1 perceived AI teaching stress was significantly and positively associated with T1 skill anxiety, replacement anxiety, ethical anxiety, and adaptation anxiety. T2 perceived AI teaching stress was also significantly and positively associated with the four anxiety dimensions at T2. The cross-time stability paths of the four anxiety dimensions were all significant, indicating that skill anxiety, replacement anxiety, ethical anxiety, and adaptation anxiety all showed longitudinal continuity during the early phase of reform. These results further support H2.

**Table 4:** Dimension-specific paths between perceived AI teaching stress and anxiety dimensions.

Model	Path	β	SE	*t*	*p*
Skill anxiety	T1_PATS → T1_SKA	0.382	0.073	5.233	0.001
	T2_PATS → T2_SKA	0.361	0.069	5.232	0.001
	T1_SKA → T2_SKA	0.497	0.084	5.917	0.001
Replacement anxiety	T1_PATS → T1_RPA	0.428	0.076	5.632	0.001
	T2_PATS → T2_RPA	0.406	0.072	5.639	0.001
	T1_RPA → T2_RPA	0.482	0.079	6.101	0.001
Ethical anxiety	T1_PATS → T1_ETA	0.413	0.074	5.581	0.001
	T2_PATS → T2_ETA	0.392	0.07	5.6	0.001
	T1_ETA → T2_ETA	0.461	0.077	5.987	0.001
Adaptation anxiety	T1_PATS → T1_ADA	0.395	0.072	5.486	0.001
	T2_PATS → T2_ADA	0.378	0.068	5.559	0.001
	T1_ADA → T2_ADA	0.455	0.078	5.833	0.001

PATS, perceived AI teaching stress; SKA, skill anxiety; RPA, replacement anxiety; ETA, ethical anxiety; ADA, adaptation anxiety. β values are standardized path coefficients. SE indicates standard error.

#### 4.5.2 Moderating effects of school support

This section tests the moderating effects of school support. It first tests H3, namely whether school support weakens the longitudinal continuity of teachers' perceived AI teaching stress from T1 to T2. It then tests H4, namely whether school support weakens the positive relationship between perceived AI teaching stress and AI teaching anxiety at T2.

Regarding H3, the overall model results are presented in Table 5. Emotional support significantly weakened the effect of T1 perceived AI teaching stress on T2 perceived AI teaching stress, β = −0.134, SE = 0.061, *T* = −2.197, *p* = 0.028. The interaction term of training support approached significance, β = −0.112, SE = 0.059, *T* = −1.898, *p* = 0.058. The interaction terms of resource support and institutional support were not statistically significant. These results indicate that emotional support reduced the extent to which stress experiences in the early phase of reform continued into the subsequent stage. Therefore, H3 was partially supported.

**Table 5:** Overall moderating effects of school support on the continuity of perceived AI teaching stress.

Interaction path	β	SE	*t*	*p*
EMS × T1_PATS → T2_PATS	−0.134	0.061	−2.197	0.028
RES × T1_PATS → T2_PATS	−0.086	0.055	−1.564	0.118
TRS × T1_PATS → T2_PATS	−0.112	0.059	−1.898	0.058
INS × T1_PATS → T2_PATS	−0.078	0.057	−1.368	0.172

PATS, perceived AI teaching stress; EMS, emotional support; RES, resource support; TRS, training support;, institutional support. β values are standardized coefficients. SE indicates standard error.

Further dimension-specific results for stress continuity are presented in Table 6. Emotional support significantly weakened the path from T1 perceived AI teaching stress to T2 perceived AI teaching stress in the skill anxiety, ethical anxiety, and replacement anxiety models. Resource support showed a significant negative moderating effect in the adaptation anxiety and replacement anxiety models. Training support showed a significant negative moderating effect in the replacement anxiety model and approached significance in the skill anxiety and adaptation anxiety models. Institutional support showed a significant negative moderating effect only in the ethical anxiety model. These results indicate that the buffering effect of school support on stress continuity varied by dimension, and that different forms of support did not weaken the cross-time continuity of teachers' stress in the same way.

**Table 6:** Dimension-specific moderating effects of school support on the continuity of perceived AI teaching stress.

Model	Interaction path	β	SE	*t*	*p*
Skill anxiety model	EMS × T1_PATS → T2_PATS	−0.129	0.059	−2.186	0.029
	RES × T1_PATS → T2_PATS	−0.082	0.053	−1.547	0.122
	TRS × T1_PATS → T2_PATS	−0.108	0.057	−1.895	0.058
	INS × T1_PATS → T2_PATS	−0.075	0.055	−1.364	0.173
Adaptation anxiety model	EMS × T1_PATS → T2_PATS	−0.095	0.06	−1.583	0.114
	RES × T1_PATS → T2_PATS	−0.122	0.056	−2.179	0.029
	TRS × T1_PATS → T2_PATS	−0.11	0.058	−1.897	0.058
	INS × T1_PATS → T2_PATS	−0.072	0.054	−1.333	0.183
Ethical anxiety model	EMS × T1_PATS → T2_PATS	−0.142	0.062	−2.29	0.022
	RES × T1_PATS → T2_PATS	−0.072	0.055	−1.309	0.191
	TRS × T1_PATS → T2_PATS	−0.106	0.058	−1.828	0.068
	INS × T1_PATS → T2_PATS	−0.129	0.057	−2.263	0.024
Replacement anxiety model	EMS × T1_PATS → T2_PATS	−0.127	0.063	−2.016	0.044
	RES × T1_PATS → T2_PATS	−0.132	0.059	−2.237	0.025
	TRS × T1_PATS → T2_PATS	−0.148	0.062	−2.387	0.017
	INS × T1_PATS → T2_PATS	−0.105	0.058	−1.81	0.07

PATS, perceived AI teaching stress; EMS, emotional support; RES, resource support; TRS, training support; INS, institutional support. β values are standardized coefficients. SE indicates standard error.

Regarding H4, the overall model results are presented in Table 7. The interaction between emotional support and T2 perceived AI teaching stress significantly and negatively predicted T2 overall AI teaching anxiety, β = −0.151, SE = 0.065, *T* = −2.323, *p* = 0.020. The interaction between training support and T2 perceived AI teaching stress was also significant, β = −0.128, SE = 0.062, *T* = −2.065, *p* = 0.039. The interaction terms of resource support and institutional support were negative in direction but did not reach statistical significance. These results indicate that, under conditions of higher emotional support and higher training support, the positive relationship between T2 perceived AI teaching stress and T2 overall AI teaching anxiety was significantly weakened. Therefore, H4 was partially supported.

**Table 7:** Overall moderating effects of school support on the association between T2 perceived AI teaching stress and T2 AI teaching anxiety.

Path	β	SE	*t*	*p*
EMS × T2_PATS → T2_ATA	−0.151	0.065	−2.323	0.02
TRS × T2_PATS → T2_ATA	−0.128	0.062	−2.065	0.039
RES × T2_PATS → T2_ATA	−0.095	0.058	−1.638	0.102
INS × T2_PATS → T2_ATA	−0.103	0.06	−1.717	0.086

PATS, perceived AI teaching stress; ATA, AI teaching anxiety; EMS, emotional support; RES, resource support; TRS, training support; INS, institutional support. β values are standardized coefficients. SE indicates standard error.

Further dimension-specific moderating effects are presented in Table 8. Emotional support showed significant negative moderating effects in the skill anxiety, ethical anxiety, and replacement anxiety models, indicating that emotional support could relatively consistently weaken the conversion of T2 perceived AI teaching stress into different types of anxiety. Training support showed significant negative moderating effects in the skill anxiety and replacement anxiety models, suggesting that training support mainly buffered the effects of stress by reducing concerns about insufficient competence and occupational replacement. Resource support showed significant negative moderating effects in the adaptation anxiety and replacement anxiety models, indicating that equipment platforms, teaching materials, and practical assistance were more helpful in alleviating teachers' anxiety about classroom integration and job replacement. Institutional support showed significant negative moderating effects in the ethical anxiety and replacement anxiety models, indicating that clear norms, evaluation standards, and organizational arrangements helped reduce uncertainty about ethical responsibility and instability in professional roles.

**Table 8:** Dimension-specific moderating effects of school support on the association between T2 perceived AI teaching stress and T2 anxiety dimensions.

Model	Interaction path	β	SE	*t*	*p*
Skill anxiety	EMS × T2_PATS → T2_SKA	−0.145	0.063	−2.302	0.021
	RES × T2_PATS → T2_SKA	−0.089	0.056	−1.589	0.112
	TRS × T2_PATS → T2_SKA	−0.121	0.06	−2.017	0.044
	INS × T2_PATS → T2_SKA	−0.094	0.058	−1.621	0.105
Adaptation anxiety	EMS × T2_PATS → T2_ADA	−0.102	0.064	−1.594	0.111
	RES × T2_PATS → T2_ADA	−0.131	0.058	−2.259	0.024
	TRS × T2_PATS → T2_ADA	−0.118	0.061	−1.934	0.053
	INS × T2_PATS → T2_ADA	−0.087	0.059	−1.475	0.14
Ethical anxiety	EMS × T2_PATS → T2_ETA	−0.155	0.064	−2.422	0.015
	RES × T2_PATS → T2_ETA	−0.079	0.057	−1.386	0.166
	TRS × T2_PATS → T2_ETA	−0.112	0.059	−1.898	0.058
	INS × T2_PATS → T2_ETA	−0.136	0.06	−2.267	0.023
Replacement anxiety	EMS × T2_PATS → T2_RPA	−0.138	0.066	−2.091	0.037
	RES × T2_PATS → T2_RPA	−0.145	0.062	−2.339	0.019
	TRS × T2_PATS → T2_RPA	−0.162	0.065	−2.492	0.013
	INS × T2_PATS → T2_RPA	−0.121	0.061	−1.984	0.047

PATS, perceived AI teaching stress; SKA, skill anxiety; ADA, adaptation anxiety; ETA, ethical anxiety; RPA, replacement anxiety; EMS, emotional support; RES, resource support; TRS, training support; INS, institutional support. β values are standardized coefficients. SE indicates standard error.

Taken together, Tables 5–8 show that the buffering effects of school support were not homogeneous. For the longitudinal continuity of stress, emotional support showed the most stable effect, while resource support, training support, and institutional support mainly functioned in specific dimension models. For the relationship between T2 stress and T2 anxiety, emotional support and training support reached statistical significance in the overall model. Further dimension-specific models showed that different types of support corresponded to different buffering paths for different anxiety dimensions. Overall, both H3 and H4 were partially supported.

### 4.6 Multigroup analysis

**Figure 6:**
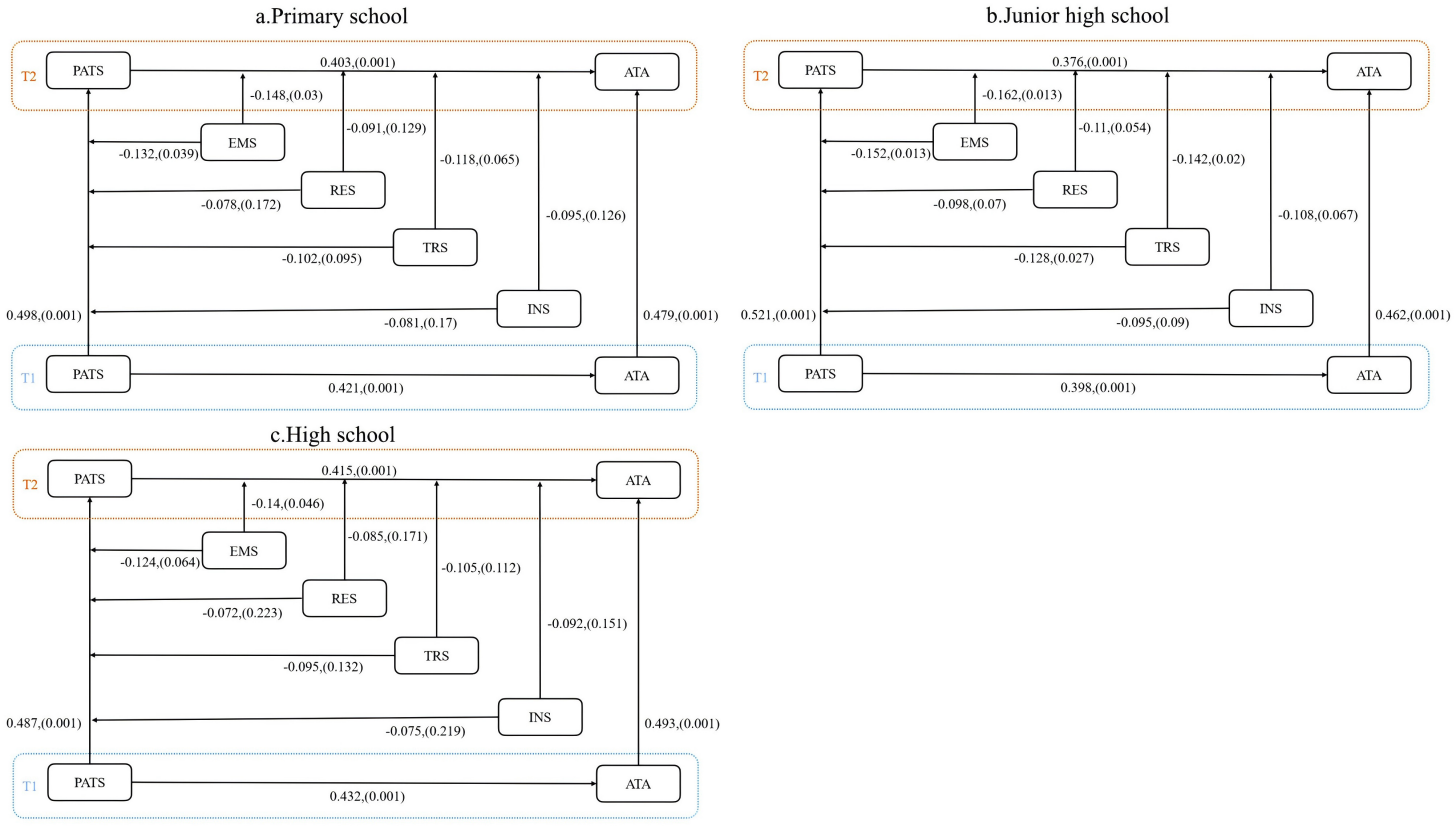
Multi-group structural equation models by school level. (**a**) Primary school; (**b**) junior high school; (**c**) senior high school. T1, Time 1; T2, Time 2; PATS, Perceived AI Teaching Stress; ATA, AI Teaching Anxiety; EMS, Emotional Support; RES, Resource Support; TRS, Training Support; INS, Institutional Support. Values are standardized path coefficients, with *p*-values reported in parentheses.

This section tests H5, namely whether the buffering effect of school support on the relationship between stress and anxiety shows differentiated patterns across teachers at different school levels. The multi-group analysis is shown in Figure 6. Detailed results are presented in Supplementary material 1, Appendix 3, Table S8. The association between perceived stress and anxiety was significant across all school levels. The path coefficient was highest among senior secondary school teachers (β = 0.415) and relatively lowest among junior secondary school teachers (β = 0.376), indicating that the effect of stress on anxiety was most pronounced among senior secondary school teachers. Regarding the moderating effects, emotional support significantly buffered the effect of stress on anxiety across all school levels. However, the moderating effect of training support was significant only among junior secondary school teachers, while the effects of resource support and institutional support did not show stable differences across school levels.

### 4.7 Qualitative analysis

To gain a deeper understanding of teachers' individual experiences of artificial intelligence-related anxiety and the contextual factors that shape it, this study conducted semi-structured interviews with 10 primary and secondary school teachers. As shown in Supplementary material 1, Appendix 4, Table S9, the interviewees included six women and four men, with teaching experience ranging from 3 to 25 years. The sample covered primary school, junior secondary school, and senior secondary school teachers and included subject backgrounds in Chinese, mathematics, English, science, information technology, and integrated practice. Anxiety levels ranged from relatively low to moderate and high.

To uncover the deeper psychological mechanisms underlying the relations identified in the structural equation models, open coding was conducted on the interview transcripts in order to capture details of the anxiety experience that quantitative data could not readily reveal and to provide contextualized explanations for the quantitative findings.

The open coding results, summarized in Supplementary material 1, Appendix 4, Table S10, presented the multidimensional structure and concrete situations of teacher anxiety in the integration of artificial intelligence into the curriculum. Four core categories emerged: skill-operation anxiety, occupational identity anxiety, ethical and safety anxiety, and instructional integration anxiety. Deficiencies in support systems further intensified anxiety, manifesting as isolation from training, lagging resources, and institutional disconnection.

These findings correspond closely to the variable dimensions in the structural equation models. The four core anxiety categories map respectively onto skill anxiety, replacement anxiety, ethical anxiety, and adaptation anxiety. More importantly, the open coding revealed the concrete mechanisms behind the quantitative moderation effects. The qualitative material showed that in-depth training reduced fears of being replaced by reshaping occupational identity. The stable moderating effect of emotional support was also corroborated in the interviews, where peer assistance and recognition from school leaders were found to significantly strengthen psychological safety. In addition, the qualitative data supplemented the school-level differences identified in the multigroup analysis: primary school teachers emphasized needs related to equipment adaptation, whereas secondary school teachers were more concerned about the absence of evaluation rules and institutional arrangements.

To integrate the open coding results more systematically and identify the logical relations among categories, axial coding was then applied to the interview texts in order to explain the deeper mechanisms revealed by the quantitative model from the perspective of teachers' lived experience.

The axial coding results, summarized in Table 9, identified six core categories: skill-operation anxiety, occupational identity anxiety, ethical and safety anxiety, instructional integration anxiety, deficiencies in support systems, and mitigating factors and coping strategies. These categories correspond closely to the four anxiety dimensions in the structural model. Skill-operation anxiety corresponds to skill anxiety, occupational identity anxiety corresponds to replacement anxiety, ethical and safety anxiety corresponds to ethical anxiety, and instructional integration anxiety corresponds to adaptation anxiety. At the same time, deficiencies in support systems and mitigating factors correspond respectively to insufficient provision and effective intervention in the four forms of school support.

**Table 9:** Axial coding.

Core category	Selective open coding	Number of initial coding nodes
Skill-operation anxiety	Technical operation barriers, technology reliability anxiety, age-related fatigue, and face-related concerns	4
Professional identity anxiety	Questioning of professional value, concerns about technological dominance, and expectations of tool practicality	3
Ethical and safety anxiety	Data privacy concerns, burden of technological errors, and lack of effectiveness evaluation	3
Teaching integration anxiety	Difficulty with classroom adaptation, policy-iteration fatigue, and time and resource constraints	3
Lack of support systems	Sense of isolation in training, institutional misalignment, and need for immediate support	3
Alleviating factors and coping strategies	Positive feedback incentives, peer mutual-support, in-depth training alleviating anxiety, and teacher-student co-learning experiences	4

The qualitative analysis further clarified the deeper mechanisms underlying the quantitative results. Skill-operation anxiety reflected insufficient self-efficacy and age-related learning fatigue, explaining why skill anxiety was highly sensitive to reform-related stress. Occupational identity anxiety highlighted teachers' concerns about a weakening of their professional value, which is highly consistent with the quantitative finding that replacement anxiety showed the largest increase among the four dimensions and was especially sensitive to the buffering effect of training support. Ethical and safety anxiety centered on data privacy and technological reliability, helping to explain why institutional support significantly moderated the pathway involving ethical anxiety. Instructional integration anxiety arose from difficulties in aligning curriculum content with classroom conditions and from pressure on time resources, which corresponds to the multigroup finding that adaptation anxiety was more prominent among primary school teachers.

### 4.8 Summary of hypothesis testing results

Table 10 summarizes the support status of the hypotheses. H1 and H2 were supported, indicating that perceived AI teaching stress and AI teaching anxiety increased significantly from T1 to T2, and that there were stable positive associations and cross-time continuity between them. H3, H4, and H5 were partially supported, suggesting that the buffering effect of school support was conditional and varied according to the type of support and school level. Overall, the findings confirmed the dynamic association between perceived AI teaching stress and AI teaching anxiety, while also showing that school support played a limited but important moderating role.

**Table 10:** Summary of hypothesis testing results.

Hypothesis	Analytical method	Main result	Conclusion
H1	Repeated-measures analysis	perceived AI teaching stress and AI teaching anxiety both increased significantly from T1 to T2.	Supported
H2	Structural equation path analysis	perceived AI teaching stress was significantly and positively associated with AI teaching anxiety at both T1 and T2, and both perceived AI teaching stress and AI teaching anxiety showed significant cross-time continuity.	Supported
H3	Structural equation path analysis	Emotional support significantly weakened the path from T1 perceived AI teaching stress to T2 perceived AI teaching stress, while the effects of other forms of support were unstable.	Partially supported
H4	Structural equation path analysis	Emotional Support and training support significantly weakened the path from T2 perceived AI teaching stress to T2 AI teaching anxiety.	Partially supported
H5	Multi-group structural equation modeling	The buffering effect of school support differed across school levels.	Partially supported

## 5 Discussion

### 5.1 Stress and multidimensional anxiety in the early stage of reform

This study found that in the early phase after the launch of reform, teachers' perceived AI teaching stress and AI teaching anxiety increased simultaneously and showed a degree of temporal continuity. This indicates that the psychological response triggered by artificial intelligence curriculum reform is better understood as a stage-based adaptation process rather than a short-lived fluctuation caused by a single event. Conservation of Resources Theory suggests that when individuals perceive important resources to be under threat, actually lost, or continuously invested without timely replenishment, stress and negative emotions are more likely to arise and may continue to develop along a chain of resource depletion (Hobfoll, 1989; Hobfoll et al., 2018).

This finding is not difficult to understand. Artificial intelligence curriculum reform imposes a set of demands on teachers simultaneously, including understanding new knowledge, designing classes, aligning assessment, and assuming responsibility for ethics and safety. These demands can consume teachers' time, energy, and cognitive resources intensively within a short period. Research on educational technology change has shown that during artificial intelligence education reform, teachers often need to cope simultaneously with knowledge updating, instructional integration, insufficient professional development provision, and the identification of ethical boundaries (Tan et al., 2025). Related reviews also indicate that rapid technological change tends to increase uncertainty, work demands, and adaptation costs, thereby intensifying teachers' experiences of anxiety and stress (Fernández-Batanero et al., 2021; Henderson and Corry, 2021). The simultaneous increase in stress and anxiety observed in this study therefore essentially reflects a stage-based adaptation pressure resulting from the growth of reform demands outpacing the replenishment of resources.

More importantly, after dividing anxiety into skill, ethical, adaptation, and replacement dimensions, this study found that reform pressure did not correspond to only one form of anxiety, but rather spread through different psychological channels. Skill anxiety mainly stemmed from competence gaps and learning burden. Ethical anxiety mainly arose from responsibility boundaries and risk concerns. Adaptation anxiety mainly resulted from mismatches among curriculum, students, equipment, and classroom procedures. Replacement anxiety more directly touched upon the stability of occupational roles and the sense of professional value. Research in adjacent fields has already noted that after artificial intelligence enters K-12 education, teachers must simultaneously confront issues of educational appropriateness, data privacy, algorithmic fairness, responsibility attribution, and role change (Adams et al., 2023; Akgun and Greenhow, 2022). This indicates that anxiety in the early phase of reform is not merely a technological issue, but rather a comprehensive psychological response triggered by changes in the structure of teaching tasks.

The interview data further supported this interpretation. What teachers most frequently mentioned was not rejection of technology itself, but concern about squeezed time, loss of classroom controllability, unclear responsibility, and evaluation pressure. This suggests that the key contradiction in the early phase of reform does not lie in whether teachers are willing to accept artificial intelligence, but in whether the teaching demands, normative pressure, and responsibility boundaries that increase within a short time exceed the carrying capacity of the resources teachers can currently mobilize (Hobfoll, 1989).

### 5.2 The buffering role of school support

This study shows that school support can buffer the transformation of stress into anxiety, but this buffering effect is not equally effective for all anxiety dimensions, nor are all forms of support equally effective. In other words, the value of school support lies more in whether it targets key pain points than in whether support intensity is uniformly high. This finding is consistent with the buffering hypothesis of social support, which proposes that the major function of support in high-stress situations is not to eliminate the source of stress directly, but to reduce the strength of the effect of stress on negative psychological outcomes (Cohen and Wills, 1985).

In light of the findings, emotional support appears to function more consistently. This means that in the early stage of reform, what teachers need first is to feel understood, seen, and recognized, so that their basic sense of psychological safety can be restored. Existing research has shown that social support from principals and school leaders can improve teachers' satisfaction of basic psychological needs and their work adjustment by reducing time pressure, minimizing ambiguity in organizational conditions, and enhancing peer support (Morska et al., 2022). Related work has also shown that in contexts of high-pressure instructional change, school administrative support is closely associated with teachers' emotional experiences and burnout (Chang et al., 2022). Emotional support is therefore not merely general comfort. Through concrete management practices such as tolerance for trial and error, public recognition, task coordination, and timely responses, it helps teachers recover a sense of control and safety.

By contrast, training support, resource support, and institutional support appear to be more targeted in nature. Training support more readily reduces skill anxiety because it can transform competence gaps into a clear and attainable learning path. Resource support more readily alleviates adaptation anxiety because equipment, teaching materials, and ready-to-use lesson examples reduce uncertainty in classroom implementation. Institutional support more readily reduces ethical anxiety because the clearer the ethical boundaries, compliance procedures, and division of responsibility, the less teachers need to repeatedly guess within gray areas. Existing studies show that school support, technological readiness, and the organizational environment significantly shape teachers' stress levels and continued adoption intentions during change (Dong et al., 2020; Joo et al., 2016). Qualitative research on the use of emerging technologies also suggests that insufficient training, inadequate school support, privacy and responsibility concerns, and role pressure are all important sources of teacher stress (Khlaif et al., 2023). In the context of artificial intelligence education, both the European Commission and UNESCO emphasize that teachers need sustained training, resource provision, and institutional ethical guidance in order to conduct artificial intelligence teaching more responsibly (European Commission, 2022; Holmes and Miao, 2023).

Accordingly, the present study does not understand school support as simply raising support levels in general, but rather as a more realistic logic of resource allocation. Under conditions of limited budgets and time, schools need to provide support in bundles and to emphasize different forms of support at different phases. Emotional support stabilizes expectations, training support fills competence gaps, resource support lowers implementation costs, and institutional support clarifies boundaries and reduces risk. Only in this way can statistical relations be translated into a management logic that is executable in the actual reform setting.

### 5.3 Differences across school levels

The multigroup results showed that teachers at different school levels differed in both the strength of the transformation from stress to anxiety and the way they responded to support. These differences are not merely demographic, but instead reflect contextual differences arising from the structure of teaching tasks and the external structure of evaluation. Artificial intelligence teaching at the primary level emphasizes initiation and experience, so teachers are more dependent on classroom activity design and the availability of equipment and are therefore more likely to experience anxiety related to curriculum adaptation and classroom implementation. Junior secondary school and senior secondary school teaching goals place greater emphasis on disciplinary structure, alignment with evaluation, and normative requirements, making teachers more likely to experience strong anxiety related to responsibility boundaries, evaluation pressure, and professional roles.

Research in adjacent areas supports this interpretation. Reviews of K-12 artificial intelligence education indicate that curriculum content, teaching methods, and the presentation of ethical issues should differ clearly across age groups (Li, 2022). Research on K-12 artificial intelligence ethics also points out that privacy, safety, fairness, and educational appropriateness do not carry the same weight across school levels (Adams et al., 2023; Akgun and Greenhow, 2022). In addition, multigroup studies have found significant differences in the mechanisms through which stress forms and in the pathways through which school support operates across instructional levels (Chou and Chou, 2021). The conclusion of this study that support strategies should be configured by school level therefore has a relatively clear theoretical and empirical basis.

## 6 Implications

### 6.1 Theoretical implications

The theoretical significance of this study lies in two main respects. First, in the context of artificial intelligence curriculum reform, the study integrates Conservation of Resources Theory and the buffering hypothesis of social support in order to explain why stress and anxiety rise simultaneously in the early stage of reform and why school support does not directly eliminate sources of stress but instead reduces the strength of the effect of stress on anxiety through a buffering mechanism. Conservation of Resources Theory emphasizes the temporality of resource gaps and resource replenishment, whereas the buffering hypothesis emphasizes the slope effect of support in high-stress situations. Together, these perspectives provide a clearer explanation of psychological fluctuations and support mechanisms in the early phase of reform (Cohen and Wills, 1985; Hobfoll, 1989).

Second, the study treats teacher anxiety as a multidimensional structure and demonstrates heterogeneity across school levels through multigroup comparison. This multidimensional approach means that anxiety is no longer treated as a generic outcome variable, but as a structured construct that can be matched to different points of intervention. Heterogeneity across school levels further indicates that the psychological consequences of the same reform and the benefits of support are not identical across teaching contexts. This moves the literature from average explanation toward layered explanation and provides a basis for future studies to construct more refined mechanistic models.

### 6.2 Practical significance

The practical significance of this study lies primarily in timing. The early stage after reform begins is the window in which stress and anxiety are most likely to rise in a concentrated way, and it is also the window in which support is most likely to generate marginal gains. If support measures are implemented only after psychological changes have already occurred, teachers are more likely to enter a state of exhaustion and withdrawal, and later resource investment will be less likely to produce positive effects. School support therefore needs to be front-loaded and advanced by stages.

The first recommendation is to make emotional support the first line of defense immediately after reform begins. Practical measures can be simple. For example, within two weeks after reform is launched, school leaders can issue a clear statement that tolerates trial teaching, trial and error, and adjustment as normal parts of the process. Teaching-research meetings can also include a regular teacher feedback session so that problems become visible and discussable. At the same time, peer-support groups can be organized by grade or subject, with a brief weekly exchange focused on sharing one reusable classroom practice or one lesson learned from difficulty. The goal is to stabilize teachers' psychological safety first and make them willing to try.

The second recommendation is to make training support the main strategic focus during the first one to three months after launch and to advance it together with institutional support. Training should not remain at the level of tool operation, but should provide transferable instructional models and lesson packages that teachers can directly use. At the institutional level, schools should simultaneously issue clear ethical and compliance checklists, for example regarding student data processing, the boundaries of artificial intelligence-generated content, and requirements for classroom assignments. This can reduce repeated guesswork in gray areas. For junior secondary school and senior secondary school teachers in particular, expectations regarding assessment, classroom goals, and ethical boundaries need to be made explicit so that uncertainty does not continue to be converted into sustained anxiety.

### 6.3 Limitations and future directions

This study still has several limitations. First, it adopted a two-wave longitudinal tracking design, which can reveal temporal associations and cross-time continuity among variables. However, because no experimental manipulation or random assignment was used, the conclusions are mainly limited to structural path relationships and longitudinal statistical associations. Future research could combine quasi-experimental designs, natural experimental designs, or more complete cross-lagged longitudinal designs to further examine the mechanisms linking artificial intelligence curriculum reform stress, school support, and teachers' anxiety.

Second, to match T1 and T2 data, this study used random codes for pseudonymous tracking rather than a fully anonymous survey. Although protective measures were adopted, including code-based matching, data de-identification, separate storage of contact information and questionnaire data, and preventing schools from accessing individual-level data, teachers in a school reform context may still be influenced by socially desirable responding and concerns about being evaluated. Future research could use independent third-party data collection, stricter anonymous tracking mechanisms, or indirect measurement methods to further reduce self-presentation bias.

Third, this study was mainly based on teachers' self-reported data. Although temporal separation, quality control procedures, and statistical tests were used to reduce the risk of common method bias, future studies could combine classroom observations, school support records, teaching-platform behavioral data, and student learning performance data to construct a multi-source evidence chain.

Fourth, the sample was drawn from regions where artificial intelligence curriculum reform had been implemented relatively early. Therefore, the applicability of the findings to other regions and different stages of reform requires further validation. Future research could expand regional coverage and continue to track teachers' psychological adaptation trajectories in the middle and later stages of reform.

## 7 Conclusion

This study examined teachers' stress and anxiety during the early phase of AI course reform and yielded three main conclusions. First, both teaching stress and teaching anxiety rose in the initial stage of reform and remained relatively continuous over time, suggesting that teachers' psychological responses should be understood as part of a stage-based adaptation process rather than as short-term fluctuations. Second, teachers' anxiety was shown to be multidimensional, encompassing skill, ethical, adaptation, and replacement anxiety. This indicates that anxiety during reform does not stem from a single source, but from distinct concerns that call for different forms of support. Third, school support played a buffering role in the link between stress and anxiety, although its effects varied across support types and school levels. Emotional support appeared to function more consistently, whereas training, resource, and institutional support were more targeted in their effects. In addition, teachers at different school levels differed in both the strength of the stress–anxiety relationship and their responsiveness to support.

Overall, the findings suggest that teachers' psychological adaptation to AI course reform is differentiated, dynamic, and context-dependent. By clarifying the longitudinal association between reform-related stress and different forms of anxiety, as well as the role of school support in weakening this association, the study provides an evidence-based foundation for more timely, targeted, and school-level-specific support strategies during the early implementation stage of reform.

## Data Availability

The raw data supporting the conclusions of this article will be made available by the authors, without undue reservation.
