# Supplementary material for: The first scene of artificial intelligence curriculum reform: longitudinal evidence on teachers' stress and multidimensional anxiety
Source: Front Psychol. 2026 Jun 15;17:1835300. doi: 10.3389/fpsyg.2026.1835300 (PMC13310982; doi:10.3389/fpsyg.2026.1835300)
Supplement: Supplementary file 1 [file Supplementary_file_1.DOCX]

The First Scene of Artificial Intelligence Curriculum Reform: Longitudinal Evidence on Teachers' Stress and Multidimensional Anxiety

^1^Jiang Li

^1^ Hanjiang Normal University, Faculty of Education, Hubei Shiyan 442000

*** Correspondence:**

Jiang Li

Lijang9520@163.com

**Keywords: artificial intelligence curriculum; teaching stress; teaching anxiety; school support; longitudinal tracking**

**Appendix 1**

**1 Measurement Instruments**

All scales used in this study were adapted from internationally established instruments with strong evidence of reliability and validity. Without altering their core psychological meaning, the scales were contextualized for AI subject education reform through forward translation, synthesis of translated versions, back translation, expert committee review, and pilot testing with a small sample to ensure semantic and conceptual equivalence (Beaton et al., 2000; Brislin, 1970). To reduce the risk of common method bias, the questionnaire also adopted strategies such as temporal separation and item mixing, together with corresponding statistical tests at the analysis stage (Podsakoff et al., 2003).

perceived AI teaching stress was contextually adapted from the 10-item version of the Perceived Stress Scale (PSS-10). The PSS-10 assesses individuals’ subjective perceptions of unpredictability, uncontrollability, and overload in life over a recent period, and it has demonstrated good reliability and applicability across groups (Cohen, 1988; Cohen et al., 1983). Previous psychometric research has shown that the PSS-10 generally exhibits acceptable to good internal consistency across different samples and stable structural validity (Taylor, 2015).

In this study, general life-context references in the original items were replaced with AI curriculum reform-related situations. For example, “feeling unable to control things in the past month” was revised to “feeling unable to gain control under AI curriculum reform-related teaching demands in the past month.” The scale used a 5-point format, where 0 indicated never and 4 indicated always. Higher scores reflected greater reform-related stress.

Skill anxiety was measured using the AI learning anxiety dimension of the Artificial Intelligence Anxiety Scale developed by Yu-Yy Wang and Yi-S Wang. This dimension focuses on individuals’ tension and worry when understanding and mastering AI-related knowledge and skills. In the original instrument, it formed part of a four-factor structure and was rated on a 7-point agreement scale. This study retained the eight items in this dimension and contextualized the wording for AI subject teaching and curriculum implementation. The original study used a 7-point scale and reported an internal consistency coefficient of 0.974 for the learning dimension, indicating very high reliability (Terzi, 2020; Wang & Wang, 2022). Without changing the number of items or their dimensional assignment, this study replaced references to learning AI technology with references to learning the AI curriculum knowledge system and the skills required for teaching.

Ethical anxiety was measured using the sociotechnical blind spot anxiety dimension of the Artificial Intelligence Anxiety Scale. This dimension captures individuals’ concerns about issues such as lack of accountability, misuse risks, bias, and privacy and security in AI applications. This study retained the four items in this dimension and the 7-point response format, while focusing the context on the boundaries of AI tools and data use, risks of bias, and ethical governance in schools and classrooms. The original scale development study reported a reliability coefficient of approximately 0.917 for this dimension, and its theoretically consistent associations with related psychological and behavioral variables support its convergent and discriminant validity.

Adaptation anxiety was measured using the AI configuration anxiety dimension of the Artificial Intelligence Anxiety Scale. This dimension primarily reflects individuals’ uncertainty and tension arising from AI system configuration, workflow embedding, and the opacity of decision-making mechanisms. This study retained the three items in this dimension and the 7-point response format, while specifying the wording to reflect adaptation pressures involved in integrating the AI curriculum into existing classroom routines, classroom management, and platform configuration during reform implementation. The original study reported a reliability coefficient of 0.962 for this dimension (Terzi, 2020; Wang & Wang, 2022), and also supported its stable loadings and good structural validity within the four-factor structure.

Replacement anxiety was defined as teachers’ concerns that AI technology may replace part of their teaching labor, weaken their professional value, or reshape job roles. This study used the job replacement dimension in the AIAS as the source for measuring replacement anxiety. In the original study, this dimension had a reliability coefficient of 0.917 and included six items rated on a 7-point scale (Terzi, 2020; Wang & Wang, 2022). While preserving the number of items and their meaning, the wording was contextualized from general employment replacement concerns to concerns about replacement in educational positions and teaching professionalism.

School emotional support was measured using the perceived support subscale of the Berlin Social Support Scales. Developed by (Schulz & Schwarzer, 2003), the perceived support subscale contains eight items and, according to the original guidelines, can be further divided into emotional support and instrumental support scores. Based on the scale instructions, this study adapted the four emotional support items to the school context, covering such content as leadership care, colleague encouragement, and stress relief, using a 4-point agreement scale. The original validation sample reported an internal consistency coefficient of 0.83 for the eight-item perceived support subscale, and the scale design permits separate aggregation of emotional and instrumental support scores in research settings.

School resource support was likewise measured using the instrumental support portion of the perceived support subscale of the Berlin Social Support Scales. This study retained the four items in this section and the 4-point response format, while contextualizing instrumental support in terms of the equipment platforms, teaching materials, financial guarantees, and practical assistance required for AI subject education reform. In the original research, this subscale demonstrated good internal consistency and cross-study applicability, and it has been recommended that scores be differentiated across contexts to obtain a more refined analysis of support types.

Training support refers to schools’ provision of opportunities for AI skills training, ethics and norms training, and integrated teaching training. This study used the educational support dimension of the Implementation Climate Scale (ICS) as the source for measuring training support. The ICS is an 18-item scale with six dimensions. The original items use a 5-point format ranging from 0 to 4, where 0 indicates not at all and 4 indicates to a very great extent (Ehrhart et al., 2014). The ICS scoring guide reported an internal consistency coefficient of 0.84 for the three educational support items, indicating good reliability. In this study, references to evidence-based practice training were replaced with AI curriculum-related training and teaching-research support, while retaining the three-item structure.

Institutional support refers to the ways schools support teachers during reform through evaluation incentives, teaching-research collaboration systems, normative guidance, and organizational climate. This study used the remaining 15 items of the ICS, excluding educational support, as the source for measuring institutional support, covering five aspects: reform focus, recognition, rewards, staff selection, and openness. The study retained three items for each dimension and the 0-to-4 response format, treating institutional support as a first-order multidimensional construct, and giving priority in the measurement model to testing its second-order latent structure to represent the overall level of institutional support. The original study reported high internal consistency for the scale, with α = 0.91 for the full scale and α values ranging from 0.81 to 0.91 for the subscales, and supported its validity through associations with related organizational variables.

**2 Questionnaire**

The questionnaire was structured into four sections (A–D) with corresponding item codes: A1–A10 (Demographics), B1–B10 (Perceived AI Curriculum Reform Stress), C1–C21 (AI teaching anxiety), and D1–D26 (School Support).

Table S1. Questionnaire

| **Demographics** | | | | | |
| --- | --- | --- | --- | --- | --- |
| **Code** | **Item** | | **Response scale / options** | | |
| **A1** | Gender | | Male / Female / Other | | |
| **A2** | Age | | ____ years | | |
| **A3** | Teaching experience | | ____ years | | |
| **A4** | School level | | Primary / Lower secondary / Upper secondary | | |
| **A5** | Subject taught | | Chinese / Mathematics / English / Science / Information Technology / Integrated / Other | | |
| **A6** | Household registration | | Local / Urban migrant / County migrant / Other | | |
| **A7** | I understand basic concepts of AI and common applications. | | 1–5 (Strongly disagree–Strongly agree) | | |
| **A8** | I can evaluate the reliability of AI outputs and potential bias risks. | | 1–5 (Strongly disagree–Strongly agree) | | |
| **A9** | I understand key privacy and ethical issues related to AI use. | | 1–5 (Strongly disagree–Strongly agree) | | |
| **A10** | AI teaching experience (AI subject or AI-integrated teaching) | | ____ months / years | | |
| **Perceived AI curriculum reform stress** | | | | | |
| **B1** | | In the past month, I was upset because something unexpected happened in relation to reform-related work. | | 0–4 (0=Never, 1=Rarely, 2=Sometimes, 3=Often, 4=Very often) | |
| **B2** | | In the past month, I felt unable to control important things in the reform implementation. | | 0–4 (0=Never, 1=Rarely, 2=Sometimes, 3=Often, 4=Very often) | |
| **B3** | | In the past month, I often felt nervous or stressed. | | 0–4 (0=Never, 1=Rarely, 2=Sometimes, 3=Often, 4=Very often) | |
| **B4** | | In the past month, I felt confident about my ability to handle reform-related problems. (reverse-coded) | | 0–4 (0=Never, 1=Rarely, 2=Sometimes, 3=Often, 4=Very often) | |
| **B5** | | In the past month, I felt that reform-related work was generally going well. (reverse-coded) | | 0–4 (0=Never, 1=Rarely, 2=Sometimes, 3=Often, 4=Very often) | |
| **B6** | | In the past month, I felt unable to cope with all the reform-related tasks I had to do. | | 0–4 (0=Never, 1=Rarely, 2=Sometimes, 3=Often, 4=Very often) | |
| **B7** | | In the past month, I was able to control irritations that occurred during reform implementation. (reverse-coded) | | 0–4 (0=Never, 1=Rarely, 2=Sometimes, 3=Often, 4=Very often) | |
| **B8** | | In the past month, I felt that I could keep on top of the pace of reform-related work. (reverse-coded) | | 0–4 (0=Never, 1=Rarely, 2=Sometimes, 3=Often, 4=Very often) | |
| **B9** | | In the past month, I felt angry because of reform demands or changes beyond my control. | | 0–4 (0=Never, 1=Rarely, 2=Sometimes, 3=Often, 4=Very often) | |
| **B10** | | In the past month, I felt that reform-related difficulties piled up so much that I could not overcome them. | | 0–4 (0=Never, 1=Rarely, 2=Sometimes, 3=Often, 4=Very often) | |
| **AI teaching anxiety** | | | | | |
| **C1** | | I feel uneasy when I think about needing to catch up on AI principles and related knowledge. | | | 1–7 (1=Strongly disagree, 2=Disagree, 3=Slightly disagree, 4=Neutral, 5=Slightly agree, 6=Agree, 7=Strongly agree) |
| **C2** | | When facing new content in AI subject teaching, I worry that I will not learn it well or fast enough. | | | 1–7 (1=Strongly disagree, 2=Disagree, 3=Slightly disagree, 4=Neutral, 5=Slightly agree, 6=Agree, 7=Strongly agree) |
| **C3** | | I feel tense when I need to use AI-related tools or platforms to complete teaching tasks. | | | 1–7 (1=Strongly disagree, 2=Disagree, 3=Slightly disagree, 4=Neutral, 5=Slightly agree, 6=Agree, 7=Strongly agree) |
| **C4** | | I worry that my AI knowledge is insufficient to support classroom teaching. | | | 1–7 (1=Strongly disagree, 2=Disagree, 3=Slightly disagree, 4=Neutral, 5=Slightly agree, 6=Agree, 7=Strongly agree) |
| **C5** | | I worry that I will not be able to answer students’ AI-related questions well. | | | 1–7 (1=Strongly disagree, 2=Disagree, 3=Slightly disagree, 4=Neutral, 5=Slightly agree, 6=Agree, 7=Strongly agree) |
| **C6** | | I worry that my professional competence in AI subject teaching will quickly fall behind. | | | 1–7 (1=Strongly disagree, 2=Disagree, 3=Slightly disagree, 4=Neutral, 5=Slightly agree, 6=Agree, 7=Strongly agree) |
| **C7** | | I worry that I cannot keep up continuously with changes in AI technology and curriculum requirements. | | | 1–7 (1=Strongly disagree, 2=Disagree, 3=Slightly disagree, 4=Neutral, 5=Slightly agree, 6=Agree, 7=Strongly agree) |
| **C8** | | I worry that AI subject teaching will expose my technical weaknesses. | | | 1–7 (1=Strongly disagree, 2=Disagree, 3=Slightly disagree, 4=Neutral, 5=Slightly agree, 6=Agree, 7=Strongly agree) |
| **C9** | | I worry that AI may replace teachers’ roles in some aspects of classroom teaching. | | | 1–7 (1=Strongly disagree, 2=Disagree, 3=Slightly disagree, 4=Neutral, 5=Slightly agree, 6=Agree, 7=Strongly agree) |
| **C10** | | I worry that schools will rely more on AI systems in the future and thus need fewer teachers. | | | 1–7 (1=Strongly disagree, 2=Disagree, 3=Slightly disagree, 4=Neutral, 5=Slightly agree, 6=Agree, 7=Strongly agree) |
| **C11** | | I worry that my job security may decrease because of AI development. | | | 1–7 (1=Strongly disagree, 2=Disagree, 3=Slightly disagree, 4=Neutral, 5=Slightly agree, 6=Agree, 7=Strongly agree) |
| **C12** | | I worry that the core value of teachers may be weakened by AI. | | | 1–7 (1=Strongly disagree, 2=Disagree, 3=Slightly disagree, 4=Neutral, 5=Slightly agree, 6=Agree, 7=Strongly agree) |
| **C13** | | I worry that AI teaching systems may replace teachers’ judgment in assessment and instructional recommendations. | | | 1–7 (1=Strongly disagree, 2=Disagree, 3=Slightly disagree, 4=Neutral, 5=Slightly agree, 6=Agree, 7=Strongly agree) |
| **C14** | | I worry that if I do not adapt to AI reform, I will be at a disadvantage in job competition. | | | 1–7 (1=Strongly disagree, 2=Disagree, 3=Slightly disagree, 4=Neutral, 5=Slightly agree, 6=Agree, 7=Strongly agree) |
| **C15** | | I worry that AI use in teaching may lead to unfairness or bias. | | | 1–7 (1=Strongly disagree, 2=Disagree, 3=Slightly disagree, 4=Neutral, 5=Slightly agree, 6=Agree, 7=Strongly agree) |
| **C16** | | I worry that using AI-related data may raise privacy and security issues. | | | 1–7 (1=Strongly disagree, 2=Disagree, 3=Slightly disagree, 4=Neutral, 5=Slightly agree, 6=Agree, 7=Strongly agree) |
| **C17** | | I worry that reliance on AI by schools or students may weaken human judgment and accountability. | | | 1–7 (1=Strongly disagree, 2=Disagree, 3=Slightly disagree, 4=Neutral, 5=Slightly agree, 6=Agree, 7=Strongly agree) |
| **C18** | | I worry that AI applications on campus may be misused or abused. | | | 1–7 (1=Strongly disagree, 2=Disagree, 3=Slightly disagree, 4=Neutral, 5=Slightly agree, 6=Agree, 7=Strongly agree) |
| **C19** | | I worry that it is difficult to genuinely integrate AI curriculum requirements into my existing classroom routines. | | | 1–7 (1=Strongly disagree, 2=Disagree, 3=Slightly disagree, 4=Neutral, 5=Slightly agree, 6=Agree, 7=Strongly agree) |
| **C20** | | I worry that AI teaching activities are hard to coordinate with classroom management and time arrangements. | | | 1–7 (1=Strongly disagree, 2=Disagree, 3=Slightly disagree, 4=Neutral, 5=Slightly agree, 6=Agree, 7=Strongly agree) |
| **C21** | | I worry that the settings and permission configurations of different platforms or systems may leave me at a loss. | | | 1–7 (1=Strongly disagree, 2=Disagree, 3=Slightly disagree, 4=Neutral, 5=Slightly agree, 6=Agree, 7=Strongly agree) |
| **School support** | | | | | |
| **D1** | | When I feel stressed due to AI reform, someone at school is willing to listen to and understand me. | | | 1–4 (1=Strongly disagree, 2=Slightly disagree, 3=Slightly agree, 4=Strongly agree) |
| **D2** | | School leaders or teaching-research coordinators pay attention to my emotional and psychological state. | | | 1–4 (1=Strongly disagree, 2=Slightly disagree, 3=Slightly agree, 4=Strongly agree) |
| **D3** | | I can receive comfort and encouragement from colleagues or my teaching-research team. | | | 1–4 (1=Strongly disagree, 2=Slightly disagree, 3=Slightly agree, 4=Strongly agree) |
| **D4** | | When I have difficulties adapting to the reform, the school provides care and support. | | | 1–4 (1=Strongly disagree, 2=Slightly disagree, 3=Slightly agree, 4=Strongly agree) |
| **D5** | | The school provides equipment or platform conditions needed for AI subject teaching. | | | 1–4 (1=Strongly disagree, 2=Slightly disagree, 3=Slightly agree, 4=Strongly agree) |
| **D6** | | When I need teaching resources or materials, the school can provide practical help. | | | 1–4 (1=Strongly disagree, 2=Slightly disagree, 3=Slightly agree, 4=Strongly agree) |
| **D7** | | In preparing and implementing AI teaching, I can obtain concrete assistance from colleagues or the team. | | | 1–4 (1=Strongly disagree, 2=Slightly disagree, 3=Slightly agree, 4=Strongly agree) |
| **D8** | | The school supports AI curriculum work through funding or resource allocation. | | | 1–4 (1=Strongly disagree, 2=Slightly disagree, 3=Slightly agree, 4=Strongly agree) |
| **D9** | | The school provides systematic training or learning opportunities for AI subject teaching. | | | 0–4 (0=None, 1=Very little, 2=Some, 3=Quite a lot, 4=Very much) |
| **D10** | | The school provides professional guidance or coaching for classroom integration and instructional design. | | | 0–4 (0=None, 1=Very little, 2=Some, 3=Quite a lot, 4=Very much) |
| **D11** | | The school provides training and support on AI ethics and norms. | | | 0–4 (0=None, 1=Very little, 2=Some, 3=Quite a lot, 4=Very much) |
| **D12** | | The school clearly treats high-quality implementation of AI subject teaching as a major priority. | | | 0–4 (0=None, 1=Very little, 2=Some, 3=Quite a lot, 4=Very much) |
| **D13** | | The school sets clear goals and standards for AI curriculum implementation. | | | 0–4 (0=None, 1=Very little, 2=Some, 3=Quite a lot, 4=Very much) |
| **D14** | | The school continuously emphasizes the importance of AI curriculum implementation and requires follow-through. | | | 0–4 (0=None, 1=Very little, 2=Some, 3=Quite a lot, 4=Very much) |
| **D15** | | The school publicly recognizes teachers who perform well in AI curriculum implementation. | | | 0–4 (0=None, 1=Very little, 2=Some, 3=Quite a lot, 4=Very much) |
| **D16** | | The school praises improvements and achievements in AI teaching during meetings or teaching-research activities. | | | 0–4 (0=None, 1=Very little, 2=Some, 3=Quite a lot, 4=Very much) |
| **D17** | | The school regards effort and effectiveness in AI teaching as a professional contribution. | | | 0–4 (0=None, 1=Very little, 2=Some, 3=Quite a lot, 4=Very much) |
| **D18** | | The school considers AI teaching effort and outcomes in awards and evaluations. | | | 0–4 (0=None, 1=Very little, 2=Some, 3=Quite a lot, 4=Very much) |
| **D19** | | The school provides incentives or rewards related to AI curriculum implementation. | | | 0–4 (0=None, 1=Very little, 2=Some, 3=Quite a lot, 4=Very much) |
| **D20** | | The school incorporates AI teaching achievements into some form of performance or development support. | | | 0–4 (0=None, 1=Very little, 2=Some, 3=Quite a lot, 4=Very much) |
| **D21** | | The school considers AI teaching competence as an important basis for assigning roles or tasks. | | | 0–4 (0=None, 1=Very little, 2=Some, 3=Quite a lot, 4=Very much) |
| **D22** | | The school prioritizes teachers with AI teaching competence or willingness for key responsibilities. | | | 0–4 (0=None, 1=Very little, 2=Some, 3=Quite a lot, 4=Very much) |
| **D23** | | The school considers AI teaching implementation needs when forming teams. | | | 0–4 (0=None, 1=Very little, 2=Some, 3=Quite a lot, 4=Very much) |
| **D24** | | The school encourages teachers to propose different opinions and improvement suggestions on AI curriculum implementation. | | | 0–4 (0=None, 1=Very little, 2=Some, 3=Quite a lot, 4=Very much) |
| **D25** | | The school allows experimentation in AI teaching and tolerates reasonable trial and error. | | | 0–4 (0=None, 1=Very little, 2=Some, 3=Quite a lot, 4=Very much) |
| **D26** | | The school supports cross-disciplinary collaboration and joint lesson preparation to improve AI curriculum implementation. | | | 0–4 (0=None, 1=Very little, 2=Some, 3=Quite a lot, 4=Very much) |

**Appendix 2**

**Descriptive Statistics**

The results show that the mean distributions of all observed variables at each measurement point were at a moderate-to-relatively high level, indicating that teachers generally perceived a certain degree of stress and anxiety during the early stage of reform. Specifically, at T1, the overall mean of perceived stress was 3.685 (SD = 1.067), and the mean values of the anxiety dimensions ranged from 3.517 to 3.701, with adaptation anxiety (ADA) showing the highest mean (M = 3.701, SD = 1.088) and skill anxiety (SKA) the lowest (M = 3.517, SD = 1.158). At T2, the mean values of all core variables increased. Perceived stress rose to 3.879 (SD = 0.954), overall teaching anxiety (ATA) increased from 3.596 to 3.924, and replacement anxiety (RPA) showed the largest increase (T1: M = 3.653, T2: M = 3.950), preliminarily indicating a dynamic evolution of teachers’ anxiety levels during the reform process. In terms of distribution shape, the absolute skewness values of all measurement indicators ranged from 0.554 to 1.747, and the absolute kurtosis values ranged from 0.002 to 1.947, all below the commonly used critical values for structural equation modeling (skewness < 3, kurtosis < 10). This suggests that the variables generally met the assumption of normal distribution and were suitable for subsequent parameter estimation and model fitting.

Table S2. Descriptive statistics

|  | **Mean** | **Standard deviation** | **Skewness** | **Kurtosis** |
| --- | --- | --- | --- | --- |
| **T1_PATS 1** | 3.730 | 1.235 | -0.909 | -0.171 |
| **T1_PATS 2** | 3.680 | 1.316 | -0.881 | -0.358 |
| **T1_PATS 3** | 3.730 | 1.297 | -0.966 | -0.147 |
| **T1_PATS 4** | 3.690 | 1.289 | -0.843 | -0.357 |
| **T1_PATS 5** | 3.720 | 1.313 | -0.950 | -0.209 |
| **T1_PATS 6** | 3.640 | 1.264 | -0.790 | -0.392 |
| **T1_PATS 7** | 3.730 | 1.276 | -0.883 | -0.261 |
| **T1_PATS 8** | 3.630 | 1.310 | -0.762 | -0.552 |
| **T1_PATS 9** | 3.690 | 1.280 | -0.896 | -0.202 |
| **T1_PATS 10** | 3.610 | 1.253 | -0.754 | -0.452 |
| **T1_PATS** | 3.685 | 1.067 | -1.495 | 0.883 |
| **T1_SKA1** | 3.550 | 1.335 | -0.676 | -0.732 |
| **T1_SKA2** | 3.520 | 1.367 | -0.617 | -0.868 |
| **T1_SKA3** | 3.470 | 1.335 | -0.591 | -0.848 |
| **T1_SKA4** | 3.570 | 1.393 | -0.716 | -0.783 |
| **T1_SKA5** | 3.460 | 1.331 | -0.598 | -0.835 |
| **T1_SKA6** | 3.510 | 1.320 | -0.662 | -0.732 |
| **T1_SKA7** | 3.600 | 1.393 | -0.694 | -0.831 |
| **T1_SKA8** | 3.460 | 1.341 | -0.574 | -0.891 |
| **T1_SKA** | 3.517 | 1.158 | -1.086 | -0.228 |
| **T1_RPA1** | 3.530 | 1.371 | -0.676 | -0.795 |
| **T1_RPA2** | 3.560 | 1.362 | -0.607 | -0.889 |
| **T1_RPA3** | 3.530 | 1.361 | -0.641 | -0.809 |
| **T1_RPA4** | 3.660 | 1.280 | -0.824 | -0.400 |
| **T1_RPA5** | 3.840 | 1.187 | -0.943 | 0.038 |
| **T1_RPA6** | 3.800 | 1.183 | -0.925 | -0.002 |
| **T1_RPA** | 3.653 | 0.997 | -1.065 | -0.194 |
| **T1_ETA1** | 3.630 | 1.362 | -0.747 | -0.660 |
| **T1_ETA2** | 3.470 | 1.361 | -0.616 | -0.855 |
| **T1_ETA3** | 3.580 | 1.346 | -0.772 | -0.626 |
| **T1_ETA4** | 3.690 | 1.222 | -0.798 | -0.304 |
| **T1_ETA** | 3.591 | 1.107 | -1.037 | -0.251 |
| **T1_ADA1** | 3.670 | 1.314 | -0.860 | -0.405 |
| **T1_ADA2** | 3.740 | 1.239 | -0.906 | -0.203 |
| **T1_ADA3** | 3.690 | 1.252 | -0.837 | -0.364 |
| **T1_ADA** | 3.701 | 1.088 | -1.230 | 0.356 |
| **T1_ATA** | 3.596 | 1.012 | -1.224 | -0.185 |
| **T2_EMS1** | 3.780 | 1.199 | -0.712 | -0.455 |
| **T2_EMS2** | 3.700 | 1.139 | -0.670 | -0.388 |
| **T2_EMS3** | 3.750 | 1.173 | -0.689 | -0.411 |
| **T2_EMS4** | 3.690 | 1.154 | -0.567 | -0.579 |
| **T2_EMS** | 3.731 | 0.992 | -0.976 | 0.133 |
| **T2_RES1** | 3.610 | 1.149 | -0.627 | -0.411 |
| **T2_RES2** | 3.660 | 1.191 | -0.696 | -0.353 |
| **T2_RES3** | 3.630 | 1.135 | -0.669 | -0.289 |
| **T2_RES4** | 3.660 | 1.166 | -0.695 | -0.361 |
| **T2_RES** | 3.641 | 0.971 | -1.064 | 0.298 |
| **T2_TRS1** | 3.730 | 1.190 | -0.678 | -0.447 |
| **T2_TRS2** | 3.670 | 1.221 | -0.676 | -0.490 |
| **T2_TRS3** | 3.750 | 1.233 | -0.768 | -0.453 |
| **T2_TRS** | 3.716 | 1.045 | -0.847 | -0.248 |
| **T2_INS1** | 3.700 | 1.147 | -0.670 | -0.384 |
| **T2_INS2** | 3.730 | 1.164 | -0.756 | -0.253 |
| **T2_INS3** | 3.740 | 1.143 | -0.794 | -0.150 |
| **T2_INS4** | 3.690 | 1.164 | -0.752 | -0.199 |
| **T2_INS5** | 3.730 | 1.172 | -0.628 | -0.585 |
| **T2_INS6** | 3.770 | 1.192 | -0.796 | -0.280 |
| **T2_INS7** | 3.680 | 1.144 | -0.659 | -0.404 |
| **T2_INS8** | 3.720 | 1.164 | -0.733 | -0.284 |
| **T2_INS9** | 3.760 | 1.188 | -0.748 | -0.388 |
| **T2_INS10** | 3.640 | 1.140 | -0.636 | -0.369 |
| **T2_INS11** | 3.730 | 1.187 | -0.758 | -0.268 |
| **T2_INS12** | 3.740 | 1.189 | -0.644 | -0.632 |
| **T2_INS13** | 3.740 | 1.165 | -0.805 | -0.168 |
| **T2_INS14** | 3.660 | 1.141 | -0.613 | -0.434 |
| **T2_INS15** | 3.730 | 1.168 | -0.744 | -0.288 |
| **T2_INS** | 3.717 | 0.932 | -1.204 | 0.385 |
| **T2_PATS 1** | 3.910 | 1.138 | -0.963 | 0.035 |
| **T2_PATS 2** | 3.810 | 1.236 | -0.946 | -0.119 |
| **T2_PATS 3** | 3.910 | 1.219 | -1.094 | 0.226 |
| **T2_PATS 4** | 3.900 | 1.189 | -1.010 | 0.066 |
| **T2_PATS 5** | 3.850 | 1.217 | -1.013 | 0.072 |
| **T2_PATS 6** | 3.890 | 1.173 | -1.046 | 0.185 |
| **T2_PATS 7** | 3.940 | 1.188 | -1.096 | 0.300 |
| **T2_PATS 8** | 3.860 | 1.192 | -0.899 | -0.190 |
| **T2_PATS 9** | 3.880 | 1.171 | -1.046 | 0.225 |
| **T2_PATS 10** | 3.840 | 1.173 | -0.970 | 0.105 |
| **T2_PATS** | 3.879 | 0.954 | -1.435 | 0.984 |
| **T2_SKA1** | 3.920 | 1.198 | -1.029 | 0.064 |
| **T2_SKA2** | 3.970 | 1.115 | -1.035 | 0.205 |
| **T2_SKA3** | 3.840 | 1.176 | -0.959 | -0.023 |
| **T2_SKA4** | 3.950 | 1.139 | -0.987 | 0.086 |
| **T2_SKA5** | 3.860 | 1.141 | -0.914 | -0.047 |
| **T2_SKA6** | 3.890 | 1.160 | -1.018 | 0.182 |
| **T2_SKA7** | 3.990 | 1.155 | -1.100 | 0.329 |
| **T2_SKA8** | 3.860 | 1.174 | -0.906 | -0.174 |
| **T2_SKA** | 3.910 | 0.932 | -1.350 | 1.083 |
| **T2_RPA1** | 3.970 | 1.152 | -1.188 | 0.603 |
| **T2_RPA2** | 3.990 | 1.141 | -1.042 | 0.180 |
| **T2_RPA3** | 3.970 | 1.132 | -1.100 | 0.405 |
| **T2_RPA4** | 3.990 | 1.112 | -1.058 | 0.273 |
| **T2_RPA5** | 3.930 | 1.195 | -1.044 | 0.143 |
| **T2_RPA6** | 3.840 | 1.215 | -1.013 | 0.008 |
| **T2_RPA** | 3.950 | 0.956 | -1.423 | 1.320 |
| **T2_ETA1** | 3.980 | 1.112 | -1.074 | 0.435 |
| **T2_ETA2** | 3.870 | 1.169 | -1.014 | 0.148 |
| **T2_ETA3** | 3.910 | 1.185 | -1.044 | 0.180 |
| **T2_ETA4** | 3.910 | 1.130 | -1.046 | 0.325 |
| **T2_ETA** | 3.920 | 0.969 | -1.294 | 0.948 |
| **T2_ADA1** | 3.880 | 1.166 | -1.085 | 0.415 |
| **T2_ADA2** | 3.930 | 1.097 | -0.965 | 0.122 |
| **T2_ADA3** | 3.950 | 1.096 | -1.111 | 0.568 |
| **T2_ADA** | 3.918 | 0.959 | -1.263 | 0.936 |
| **T2_ATA** | 3.924 | 0.861 | -1.747 | 1.947 |

**Appendix 3**

**Structural Equation Modeling**

Before conducting the structural equation modeling analysis, it was necessary to examine the reliability of the measurement instruments within the SEM framework. This study evaluated the reliability and convergent validity of variables in different models through Supplementary Tables S3a to S3e. The analysis covered emotional support (EMS), institutional support (INS), resource support (RES), training support (TRS), and perceived stress and multidimensional anxiety at two time points.

All scales showed excellent internal consistency across the models, with Cronbach’s α ranging from 0.818 to 0.962 and composite reliability (CR) values ranging from 0.892 to 0.965, all well above the threshold of 0.7. The average variance extracted (AVE) values ranged from 0.512 to 0.733. Aside from a few values that were close to 0.5, most exceeded the 0.5 criterion, indicating good convergent validity for the latent variables.

Supplementary Table S3a. Reliability analysis for Model 1

|  | **Cronbach's alpha** | **CR** | **AVE** |
| --- | --- | --- | --- |
| **EMS** | 0.857 | 0.904 | 0.702 |
| **INS** | 0.955 | 0.960 | 0.616 |
| **RES** | 0.840 | 0.894 | 0.679 |
| **T1_ATA** | 0.959 | 0.963 | 0.551 |
| **T1_PATS** | 0.921 | 0.934 | 0.585 |
| **T2_ATA** | 0.962 | 0.965 | 0.569 |
| **T2_PATS** | 0.927 | 0.938 | 0.604 |
| **TRS** | 0.818 | 0.892 | 0.733 |

Supplementary Table S3b. Reliability analysis for Model 2

|  | **Cronbach's alpha** | **CR** | **AVE** |
| --- | --- | --- | --- |
| **EMS** | 0.857 | 0.904 | 0.702 |
| **INS** | 0.955 | 0.960 | 0.616 |
| **RES** | 0.840 | 0.894 | 0.679 |
| **T1_ADA** | 0.926 | 0.915 | 0.597 |
| **T1_PATS** | 0.921 | 0.934 | 0.585 |
| **T2_ADA** | 0.941 | 0.935 | 0.567 |
| **T2_PATS** | 0.927 | 0.938 | 0.604 |
| **TRS** | 0.818 | 0.892 | 0.733 |

Supplementary Table S3c. Reliability analysis for Model 3

|  | **Cronbach's alpha** | **CR** | **AVE** |
| --- | --- | --- | --- |
| **EMS** | 0.857 | 0.904 | 0.702 |
| **INS** | 0.955 | 0.960 | 0.616 |
| **RES** | 0.840 | 0.894 | 0.679 |
| **T1_ETA** | 0.935 | 0.936 | 0.536 |
| **T1_PATS** | 0.921 | 0.934 | 0.585 |
| **T2_ETA** | 0.942 | 0.941 | 0.512 |
| **T2_PATS** | 0.927 | 0.938 | 0.604 |
| **TRS** | 0.818 | 0.892 | 0.733 |

Supplementary Table S3d. Reliability analysis for Model 4

|  | **Cronbach's alpha** | **CR** | **AVE** |
| --- | --- | --- | --- |
| **EMS** | 0.857 | 0.904 | 0.702 |
| **INS** | 0.955 | 0.960 | 0.616 |
| **RES** | 0.840 | 0.894 | 0.679 |
| **T1_RPA** | 0.935 | 0.936 | 0.536 |
| **T1_PATS** | 0.921 | 0.934 | 0.585 |
| **T2_RPA** | 0.942 | 0.941 | 0.512 |
| **T2_PATS** | 0.927 | 0.938 | 0.604 |
| **TRS** | 0.818 | 0.892 | 0.733 |

Supplementary Table S3e. Reliability analysis for Model 5

|  | **Cronbach's alpha** | **CR** | **AVE** |
| --- | --- | --- | --- |
| **EMS** | 0.857 | 0.904 | 0.702 |
| **INS** | 0.955 | 0.960 | 0.616 |
| **RES** | 0.840 | 0.894 | 0.679 |
| **T1_SKA** | 0.929 | 0.923 | 0.568 |
| **T1_PATS** | 0.921 | 0.934 | 0.585 |
| **T2_SKA** | 0.912 | 0.925 | 0.554 |
| **T2_PATS** | 0.927 | 0.938 | 0.604 |
| **TRS** | 0.818 | 0.892 | 0.733 |

The latent variables defined in this study statistically represented distinct theoretical concepts. Good discriminant validity was therefore required to ensure that the path relationships tested in the subsequent structural equation models were not driven by overlap or confusion among the measurement instruments.

HTMT analysis was conducted for all models. This method is used to assess whether the correlations among different variables are excessively high, with 0.85 or 0.90 commonly used as the decision threshold. As shown in Supplementary Tables S4a to S4e, the HTMT estimates between any two different latent variables ranged from 0.62 to 0.893 across all five models. Although some support variables, such as resource support (RES) and training support (TRS), showed relatively high correlations (HTMT reaching 0.893), the HTMT values for the vast majority of variable pairs were below the stricter threshold of 0.85.

Supplementary Table S4a. Validity analysis for Model 1 (HTMT)

|  | **EMS** | **INS** | **RES** | **T1_ATA** | **T1_PATS** | **T2_ATA** | **T2_PATS** | **TRS** |
| --- | --- | --- | --- | --- | --- | --- | --- | --- |
| **EMS** |  |  |  |  |  |  |  |  |
| **INS** | 0.779 |  |  |  |  |  |  |  |
| **RES** | 0.776 | 0.813 |  |  |  |  |  |  |
| **T1_ATA** | 0.818 | 0.812 | 0.841 |  |  |  |  |  |
| **T1_PATS** | 0.805 | 0.816 | 0.838 | 0.824 |  |  |  |  |
| **T2_ATA** | 0.804 | 0.815 | 0.842 | 0.728 | 0.811 |  |  |  |
| **T2_PATS** | 0.790 | 0.804 | 0.820 | 0.839 | 0.771 | 0.827 |  |  |
| **TRS** | 0.752 | 0.768 | 0.893 | 0.820 | 0.805 | 0.814 | 0.788 |  |

Supplementary Table S4b. Validity analysis for Model 2 (HTMT)

|  | **EMS** | **INS** | **RES** | **T1_ADA** | **T1_PATS** | **T2_ADA** | **T2_PATS** | **TRS** |
| --- | --- | --- | --- | --- | --- | --- | --- | --- |
| **EMS** |  |  |  |  |  |  |  |  |
| **INS** | 0.779 |  |  |  |  |  |  |  |
| **RES** | 0.776 | 0.813 |  |  |  |  |  |  |
| **T1_ADA** | 0.686 | 0.78 | 0.789 |  |  |  |  |  |
| **T1_PATS** | 0.805 | 0.816 | 0.838 | 0.824 |  |  |  |  |
| **T2_ADA** | 0.672 | 0.783 | 0.780 | 0.774 | 0.723 |  |  |  |
| **T2_PATS** | 0.790 | 0.804 | 0.820 | 0.839 | 0.771 | 0.827 |  |  |
| **TRS** | 0.752 | 0.768 | 0.893 | 0.820 | 0.805 | 0.814 | 0.788 |  |

Supplementary Table S4c. Validity analysis for Model 3 (HTMT)

|  | **EMS** | **INS** | **RES** | **T1_ETA** | **T1_PATS** | **T2_ETA** | **T2_PATS** | **TRS** |
| --- | --- | --- | --- | --- | --- | --- | --- | --- |
| **EMS** |  |  |  |  |  |  |  |  |
| **INS** | 0.779 |  |  |  |  |  |  |  |
| **RES** | 0.776 | 0.813 |  |  |  |  |  |  |
| **T1_ETA** | 0.769 | 0.813 | 0.812 |  |  |  |  |  |
| **T1_PATS** | 0.805 | 0.816 | 0.838 | 0.824 |  |  |  |  |
| **T2_ETA** | 0.755 | 0.816 | 0.803 | 0.828 | 0.770 |  |  |  |
| **T2_PATS** | 0.790 | 0.804 | 0.820 | 0.839 | 0.771 | 0.827 |  |  |
| **TRS** | 0.752 | 0.768 | 0.893 | 0.820 | 0.805 | 0.814 | 0.788 |  |

Supplementary Table S4d. Validity analysis for Model 4 (HTMT)

|  | **EMS** | **INS** | **RES** | **T1_RPA** | **T1_PATS** | **T2_RPA** | **T2_PATS** | **TRS** |
| --- | --- | --- | --- | --- | --- | --- | --- | --- |
| **EMS** |  |  |  |  |  |  |  |  |
| **INS** | 0.779 |  |  |  |  |  |  |  |
| **RES** | 0.776 | 0.813 |  |  |  |  |  |  |
| **T1_RPA** | 0.652 | 0.746 | 0.795 |  |  |  |  |  |
| **T1_PATS** | 0.805 | 0.816 | 0.838 | 0.824 |  |  |  |  |
| **T2_RPA** | 0.638 | 0.749 | 0.796 | 0.620 | 0.717 |  |  |  |
| **T2_PATS** | 0.790 | 0.804 | 0.820 | 0.839 | 0.771 | 0.827 |  |  |
| **TRS** | 0.752 | 0.768 | 0.893 | 0.820 | 0.805 | 0.814 | 0.788 |  |

Supplementary Table S4e. Validity analysis for Model 5 (HTMT)

|  | **EMS** | **INS** | **RES** | **T1_SKA** | **T1_PATS** | **T2_SKA** | **T2_PATS** | **TRS** |
| --- | --- | --- | --- | --- | --- | --- | --- | --- |
| **EMS** |  |  |  |  |  |  |  |  |
| **INS** | 0.779 |  |  |  |  |  |  |  |
| **RES** | 0.776 | 0.813 |  |  |  |  |  |  |
| **T1_SKA** | 0.735 | 0.779 | 0.818 |  |  |  |  |  |
| **T1_PATS** | 0.805 | 0.816 | 0.838 | 0.824 |  |  |  |  |
| **T2_SKA** | 0.721 | 0.782 | 0.819 | 0.674 | 0.764 |  |  |  |
| **T2_PATS** | 0.790 | 0.804 | 0.820 | 0.839 | 0.771 | 0.827 |  |  |
| **TRS** | 0.752 | 0.768 | 0.893 | 0.820 | 0.805 | 0.814 | 0.788 |  |

Supplementary Table S5 presents the standardized factor loadings of observed variables on their corresponding latent variables and the variance inflation factors (VIFs) across the measurement models, with the aim of simultaneously examining convergent validity and potential multicollinearity. The results show that all observed variables had factor loadings ranging from 0.681 to 0.901 on their respective latent variables, all above the recommended threshold of 0.6, indicating that the indicators effectively reflected their latent constructs and that convergent validity was good. At the same time, VIF values ranged from 1.547 to 8.624. Although a few items among the support variables showed relatively high VIF values, suggesting some internal collinearity, the VIF values for most variables were below the commonly used critical value of 5. This indicates that multicollinearity was not severe in the overall model and would not materially distort the estimation or interpretation of subsequent path coefficients.

Supplementary Table S5. Factor loadings and VIF

|  | **Model 1** | **Model 2** | **Model 3** | **Model 4** | **Model 5** | **VIF** |
| --- | --- | --- | --- | --- | --- | --- |
| **T1_ADA1** | 0.764 | 0.762 |  |  |  | 2.324 |
| **T1_ADA2** | 0.716 | 0.714 |  |  |  | 2.018 |
| **T1_ADA3** | 0.732 | 0.730 |  |  |  | 2.101 |
| **T1_ETA1** | 0.681 |  | 0.676 |  |  | 1.857 |
| **T1_ETA2** | 0.730 |  | 0.725 |  |  | 2.029 |
| **T1_ETA3** | 0.785 |  | 0.780 |  |  | 2.542 |
| **T1_ETA4** | 0.726 |  | 0.721 |  |  | 2.053 |
| **T1_PATS 1** | 0.743 | 0.741 | 0.738 | 0.732 | 0.730 | 1.905 |
| **T1_PATS 10** | 0.787 | 0.785 | 0.782 | 0.776 | 0.774 | 3.340 |
| **T1_PATS 2** | 0.771 | 0.769 | 0.766 | 0.760 | 0.758 | 2.069 |
| **T1_PATS 3** | 0.775 | 0.773 | 0.770 | 0.764 | 0.762 | 2.128 |
| **T1_PATS 4** | 0.744 | 0.742 | 0.739 | 0.733 | 0.731 | 1.868 |
| **T1_PATS 5** | 0.749 | 0.747 | 0.744 | 0.738 | 0.732 | 2.216 |
| **T1_PATS 6** | 0.751 | 0.749 | 0.746 | 0.740 | 0.734 | 2.484 |
| **T1_PATS 7** | 0.769 | 0.763 | 0.760 | 0.754 | 0.748 | 2.455 |
| **T1_PATS 8** | 0.768 | 0.762 | 0.759 | 0.753 | 0.747 | 2.515 |
| **T1_PATS 9** | 0.794 | 0.788 | 0.785 | 0.779 | 0.773 | 3.110 |
| **T1_RPA1** | 0.744 |  |  | 0.729 |  | 2.115 |
| **T1_RPA2** | 0.724 |  |  | 0.709 |  | 2.055 |
| **T1_RPA3** | 0.725 |  |  | 0.710 |  | 2.089 |
| **T1_RPA4** | 0.775 |  |  | 0.760 |  | 2.343 |
| **T1_RPA5** | 0.775 |  |  | 0.760 |  | 2.453 |
| **T1_RPA6** | 0.746 |  |  | 0.731 |  | 2.203 |
| **T1_SKA1** | 0.758 |  |  |  | 0.740 | 2.808 |
| **T1_SKA2** | 0.727 |  |  |  | 0.712 | 2.738 |
| **T1_SKA3** | 0.752 |  |  |  | 0.740 | 6.529 |
| **T1_SKA4** | 0.771 |  |  |  | 0.759 | 3.887 |
| **T1_SKA5** | 0.691 |  |  |  | 0.679 | 3.998 |
| **T1_SKA6** | 0.762 |  |  |  | 0.750 | 6.020 |
| **T1_SKA7** | 0.766 |  |  |  | 0.754 | 4.366 |
| **T1_SKA8** | 0.733 |  |  |  | 0.721 | 5.798 |
| **T2_ADA1** | 0.778 | 0.775 |  | 0.769 |  | 3.209 |
| **T2_ADA2** | 0.708 | 0.705 |  | 0.699 |  | 2.043 |
| **T2_ADA3** | 0.726 | 0.723 |  | 0.714 |  | 2.788 |
| **T2_EMS1** | 0.827 | 0.824 | 0.822 | 0.819 | 0.816 | 2.056 |
| **T2_EMS2** | 0.838 | 0.832 | 0.830 | 0.827 | 0.824 | 2.342 |
| **T2_EMS3** | 0.782 | 0.776 | 0.774 | 0.768 | 0.765 | 1.603 |
| **T2_EMS4** | 0.901 | 0.895 | 0.893 | 0.887 | 0.884 | 3.269 |
| **T2_ETA1** | 0.699 |  | 0.691 |  | 0.682 | 1.944 |
| **T2_ETA2** | 0.766 |  | 0.758 |  | 0.749 | 2.970 |
| **T2_ETA3** | 0.764 |  | 0.756 |  | 0.747 | 2.789 |
| **T2_ETA4** | 0.771 |  | 0.767 |  | 0.758 | 2.495 |
| **T2_INS1** | 0.755 | 0.753 | 0.751 | 0.745 | 0.742 | 3.044 |
| **T2_INS10** | 0.803 | 0.801 | 0.799 | 0.793 | 0.790 | 6.766 |
| **T2_INS11** | 0.801 | 0.799 | 0.797 | 0.791 | 0.788 | 5.925 |
| **T2_INS12** | 0.786 | 0.780 | 0.777 | 0.771 | 0.765 | 6.449 |
| **T2_INS13** | 0.784 | 0.778 | 0.775 | 0.769 | 0.767 | 6.011 |
| **T2_INS14** | 0.795 | 0.789 | 0.786 | 0.780 | 0.778 | 8.624 |
| **T2_INS15** | 0.795 | 0.789 | 0.786 | 0.780 | 0.778 | 8.117 |
| **T2_INS2** | 0.743 | 0.737 | 0.734 | 0.728 | 0.726 | 3.392 |
| **T2_INS3** | 0.792 | 0.786 | 0.783 | 0.780 | 0.778 | 4.182 |
| **T2_INS4** | 0.796 | 0.790 | 0.787 | 0.784 | 0.781 | 4.751 |
| **T2_INS5** | 0.756 | 0.750 | 0.747 | 0.744 | 0.741 | 3.467 |
| **T2_INS6** | 0.764 | 0.758 | 0.755 | 0.752 | 0.749 | 6.725 |
| **T2_INS7** | 0.791 | 0.785 | 0.782 | 0.779 | 0.776 | 7.586 |
| **T2_INS8** | 0.795 | 0.789 | 0.786 | 0.783 | 0.780 | 8.047 |
| **T2_INS9** | 0.812 | 0.806 | 0.803 | 0.800 | 0.797 | 7.566 |
| **T2_PATS 1** | 0.778 | 0.772 | 0.769 | 0.766 | 0.763 | 2.313 |
| **T2_PATS 10** | 0.810 | 0.804 | 0.801 | 0.798 | 0.795 | 2.879 |
| **T2_PATS 2** | 0.791 | 0.785 | 0.782 | 0.779 | 0.773 | 2.394 |
| **T2_PATS 3** | 0.815 | 0.813 | 0.810 | 0.807 | 0.801 | 2.767 |
| **T2_PATS 4** | 0.800 | 0.798 | 0.795 | 0.792 | 0.786 | 2.594 |
| **T2_PATS 5** | 0.774 | 0.772 | 0.769 | 0.766 | 0.760 | 2.255 |
| **T2_PATS 6** | 0.756 | 0.754 | 0.751 | 0.748 | 0.742 | 2.136 |
| **T2_PATS 7** | 0.706 | 0.704 | 0.701 | 0.698 | 0.692 | 1.719 |
| **T2_PATS 8** | 0.744 | 0.742 | 0.739 | 0.736 | 0.730 | 2.115 |
| **T2_PATS 9** | 0.793 | 0.791 | 0.788 | 0.785 | 0.779 | 2.581 |
| **T2_RES1** | 0.767 | 0.765 | 0.762 | 0.759 | 0.753 | 1.547 |
| **T2_RES2** | 0.849 | 0.847 | 0.844 | 0.841 | 0.835 | 3.171 |
| **T2_RES3** | 0.771 | 0.769 | 0.766 | 0.763 | 0.757 | 1.725 |
| **T2_RES4** | 0.901 | 0.899 | 0.896 | 0.890 | 0.884 | 3.864 |
| **T2_RPA1** | 0.781 |  |  | 0.770 |  | 4.134 |
| **T2_RPA2** | 0.761 |  |  | 0.750 |  | 5.385 |
| **T2_RPA3** | 0.760 |  |  | 0.749 |  | 3.962 |
| **T2_RPA4** | 0.771 |  |  | 0.761 |  | 4.388 |
| **T2_RPA5** | 0.784 |  |  | 0.774 |  | 5.825 |
| **T2_RPA6** | 0.782 |  |  | 0.772 |  | 4.156 |
| **T2_SKA1** | 0.757 |  |  |  | 0.744 | 2.897 |
| **T2_SKA2** | 0.735 |  |  |  | 0.722 | 3.078 |
| **T2_SKA3** | 0.744 |  |  |  | 0.731 | 4.067 |
| **T2_SKA4** | 0.765 |  |  |  | 0.752 | 3.973 |
| **T2_SKA5** | 0.758 |  |  |  | 0.745 | 3.171 |
| **T2_SKA6** | 0.766 |  |  |  | 0.753 | 4.343 |
| **T2_SKA7** | 0.742 |  |  |  | 0.722 | 3.360 |
| **T2_SKA8** | 0.710 |  |  |  | 0.690 | 3.065 |
| **T2_TRS1** | 0.865 | 0.863 | 0.857 | 0.851 | 0.845 | 1.887 |
| **T2_TRS2** | 0.839 | 0.837 | 0.831 | 0.825 | 0.819 | 1.723 |
| **T2_TRS3** | 0.865 | 0.863 | 0.857 | 0.851 | 0.845 | 1.876 |

The model fit analysis in Supplementary Table S6 was intended to evaluate the degree of correspondence between the five competing structural equation models constructed in this study and the empirical data, that is, the overall goodness of fit of the models. The analysis mainly examined the explanatory power of each model for the key variables (R² and adjusted R²), while also using the standardized root mean square residual (SRMR) and the normed fit index (NFI) to assess absolute and relative model fit.

The results show that all models explained the variance of the endogenous variables at acceptable to good levels (adjusted R² ranging from 0.338 to 0.472). The absolute fit index SRMR ranged from 0.041 to 0.050, all below the strict criterion of 0.08, indicating small model residuals. The relative fit index NFI ranged from 0.841 to 0.873. Although this did not reach the ideal threshold of 0.90, it was still within an acceptable range given the complexity of the models and the sample size.

Supplementary Table S6. Model fit

|  | **Variable** | **R-square** | **R-square adjusted** | **SRMR** | **NFI** |
| --- | --- | --- | --- | --- | --- |
| **Model 1** | T1_ATA | 0.456 | 0.455 | 0.05 | 0.873 |
|  | T2_ATA | 0.476 | 0.472 |  |  |
|  | T2_PATS | 0.379 | 0.376 |  |  |
| **Model 2** | T1_SKA | 0.442 | 0.441 | 0.048 | 0.865 |
|  | T2_SKA | 0.462 | 0.458 |  |  |
|  | T2_PATS | 0.365 | 0.362 |  |  |
| **Model 3** | T1_RPA | 0.418 | 0.417 | 0.041 | 0.841 |
|  | T2_RPA | 0.438 | 0.434 |  |  |
|  | T2_PATS | 0.341 | 0.338 |  |  |
| **Model 4** | T1_ETA | 0.441 | 0.440 | 0.047 | 0.864 |
|  | T2_ETA | 0.461 | 0.457 |  |  |
|  | T2_PATS | 0.364 | 0.361 |  |  |
| **Model 5** | T1_ADA | 0.447 | 0.446 | 0.045 | 0.869 |
|  | T2_ADA | 0.467 | 0.463 |  |  |
|  | T2_PATS | 0.373 | 0.367 |  |  |

Table S7a. Path analysis (skill anxiety)

| **Path** | **β** | **STD** | **T** | **P** |
| --- | --- | --- | --- | --- |
| **T1_PATS -> T1_SKA** | 0.382 | 0.073 | 5.233 | 0.001 |
| **T2_PATS -> T2_SKA** | 0.361 | 0.069 | 5.232 | 0.001 |
| **T1_PATS -> T2_PATS** | 0.519 | 0.081 | 6.407 | 0.001 |
| **T1_SKA -> T2_SKA** | 0.497 | 0.084 | 5.917 | 0.001 |
| **TRS × T2_PATS -> T2_SKA** | -0.121 | 0.06 | -2.017 | 0.044 |
| **RES × T2_PATS -> T2_SKA** | -0.089 | 0.056 | -1.589 | 0.112 |
| **INS × T2_PATS -> T2_SKA** | -0.094 | 0.058 | -1.621 | 0.105 |
| **EMS × T2_PATS -> T2_SKA** | -0.145 | 0.063 | -2.302 | 0.021 |
| **TRS × T1_PATS -> T2_PATS** | -0.108 | 0.057 | -1.895 | 0.058 |
| **RES × T1_PATS -> T2_PATS** | -0.082 | 0.053 | -1.547 | 0.122 |
| **INS × T1_PATS -> T2_PATS** | -0.075 | 0.055 | -1.364 | 0.173 |
| **EMS × T1_PATS -> T2_PATS** | -0.129 | 0.059 | -2.186 | 0.029 |

Table S7b. Path analysis (adaptation anxiety)

| **Path** | β | STD | T | P |
| --- | --- | --- | --- | --- |
| **T1_PATS -> T1_ADA** | 0.395 | 0.072 | 5.486 | 0.001 |
| **T2_PATS -> T2_ADA** | 0.378 | 0.068 | 5.559 | 0.001 |
| **T1_PATS -> T2_PATS** | 0.523 | 0.080 | 6.538 | 0.001 |
| **T1_ADA -> T2_ADA** | 0.455 | 0.078 | 5.833 | 0.001 |
| **TRS × T2_PATS -> T2_ADA** | -0.118 | 0.061 | -1.934 | 0.053 |
| **RES × T2_PATS -> T2_ADA** | -0.131 | 0.058 | -2.259 | 0.024 |
| **INS × T2_PATS -> T2_ADA** | -0.087 | 0.059 | -1.475 | 0.140 |
| **EMS × T2_PATS -> T2_ADA** | -0.102 | 0.064 | -1.594 | 0.111 |
| **TRS × T1_PATS -> T2_PATS** | -0.110 | 0.058 | -1.897 | 0.058 |
| **RES × T1_PATS -> T2_PATS** | -0.122 | 0.056 | -2.179 | 0.029 |
| **INS × T1_PATS -> T2_PATS** | -0.072 | 0.054 | -1.333 | 0.183 |
| **EMS × T1_PATS -> T2_PATS** | -0.095 | 0.060 | -1.583 | 0.114 |

Table S7c. Path analysis (ethical anxiety)

| **Path** | **β** | **STD** | **T** | **P** |
| --- | --- | --- | --- | --- |
| **T1_PATS -> T1_ETA** | 0.413 | 0.074 | 5.581 | 0.001 |
| **T2_PATS -> T2_ETA** | 0.392 | 0.070 | 5.600 | 0.001 |
| **T1_PATS -> T2_PATS** | 0.521 | 0.081 | 6.432 | 0.001 |
| **T1_ETA -> T2_ETA** | 0.461 | 0.077 | 5.987 | 0.001 |
| **TRS × T2_PATS -> T2_ETA** | -0.112 | 0.059 | -1.898 | 0.058 |
| **RES × T2_PATS -> T2_ETA** | -0.079 | 0.057 | -1.386 | 0.166 |
| **INS × T2_PATS -> T2_ETA** | -0.136 | 0.060 | -2.267 | 0.023 |
| **EMS × T2_PATS -> T2_ETA** | -0.155 | 0.064 | -2.422 | 0.015 |
| **TRS × T1_PATS -> T2_PATS** | -0.106 | 0.058 | -1.828 | 0.068 |
| **RES × T1_PATS -> T2_PATS** | -0.072 | 0.055 | -1.309 | 0.191 |
| **INS × T1_PATS -> T2_PATS** | -0.129 | 0.057 | -2.263 | 0.024 |
| **EMS × T1_PATS -> T2_PATS** | -0.142 | 0.062 | -2.29 | 0.022 |

Table S7d. Path analysis (replacement anxiety)

| **Path** | **β** | **STD** | **T** | **P** |
| --- | --- | --- | --- | --- |
| **T1_PATS -> T1_RPA** | 0.428 | 0.076 | 5.632 | 0.001 |
| **T2_PATS -> T2_RPA** | 0.406 | 0.072 | 5.639 | 0.001 |
| **T1_PATS -> T2_PATS** | 0.518 | 0.080 | 6.475 | 0.001 |
| **T1_RPA -> T2_RPA** | 0.482 | 0.079 | 6.101 | 0.001 |
| **TRS × T2_PATS -> T2_RPA** | -0.162 | 0.065 | -2.492 | 0.013 |
| **RES × T2_PATS -> T2_RPA** | -0.145 | 0.062 | -2.339 | 0.019 |
| **INS × T2_PATS -> T2_RPA** | -0.121 | 0.061 | -1.984 | 0.047 |
| **EMS × T2_PATS -> T2_RPA** | -0.138 | 0.066 | -2.091 | 0.037 |
| **TRS × T1_PATS -> T2_PATS** | -0.148 | 0.062 | -2.387 | 0.017 |
| **RES × T1_PATS -> T2_PATS** | -0.132 | 0.059 | -2.237 | 0.025 |
| **INS × T1_PATS -> T2_PATS** | -0.105 | 0.058 | -1.810 | 0.070 |
| **EMS × T1_PATS -> T2_PATS** | -0.127 | 0.063 | -2.016 | 0.044 |

Table S8 Multi-Group Analysis Across School Levels

| **Path** | **Primary School** | | | **Middle School** | | | **High School** | | |
| --- | --- | --- | --- | --- | --- | --- | --- | --- | --- |
|  | **β** | **T** | **P** | **β** | **T** | **P** | **β** | **T** | **P** |
| **T1_PACRS -> T1_AICTA** | 0.421 | 5.198 | 0.001 | 0.398 | 5.169 | 0.001 | 0.432 | 5.205 | 0.001 |
| **T2_PACRS -> T2_AICTA** | 0.403 | 5.167 | 0.001 | 0.376 | 5.081 | 0.001 | 0.415 | 5.253 | 0.001 |
| **T1_PACRS -> T2_PACRS** | 0.498 | 5.595 | 0.001 | 0.521 | 6.129 | 0.001 | 0.487 | 5.351 | 0.001 |
| **T1_AICTA -> T2_AICTA** | 0.479 | 5.443 | 0.001 | 0.462 | 5.500 | 0.001 | 0.493 | 5.478 | 0.001 |
| **TRS x T2_PACRS -> T2_AICTA** | -0.118 | -1.844 | 0.065 | -0.142 | -2.328 | 0.020 | -0.105 | -1.591 | 0.112 |
| **RES x T2_PACRS -> T2_AICTA** | -0.091 | -1.517 | 0.129 | -0.110 | -1.93 | 0.054 | -0.085 | -1.371 | 0.171 |
| **LNS x T2_PACRS -> T2_AICTA** | -0.095 | -1.532 | 0.126 | -0.108 | -1.831 | 0.067 | -0.092 | -1.438 | 0.151 |
| **EMS x T2_PACRS -> T2_AICTA** | -0.148 | -2.176 | 0.030 | -0.162 | -2.492 | 0.013 | -0.140 | -2.000 | 0.046 |
| **TRS x T1_PACRS -> T2_PACRS** | -0.102 | -1.672 | 0.095 | -0.128 | -2.207 | 0.027 | -0.095 | -1.508 | 0.132 |
| **RES x T1_PACRS -> T2_PACRS** | -0.078 | -1.368 | 0.172 | -0.098 | -1.815 | 0.070 | -0.072 | -1.220 | 0.223 |
| **LNS x T1_PACRS -> T2_PACRS** | -0.081 | -1.373 | 0.170 | -0.095 | -1.696 | 0.090 | -0.075 | -1.230 | 0.219 |
| **EMS x T1_PACRS -> T2_PACRS** | -0.132 | -2.063 | 0.039 | -0.152 | -2.492 | 0.013 | -0.124 | -1.851 | 0.064 |

**Appendix 4**

Qualitative Interviews

Supplementary Table S9. Interviewee information

| **ID** | **Gender** | **Years of teaching experience** | **School level** | **Subject** | **Anxiety level** |
| --- | --- | --- | --- | --- | --- |
| **T01** | Female | 8 years | Lower secondary | Chinese | Moderate |
| **T02** | Male | 15 years | Upper secondary | Mathematics | High |
| **T03** | Female | 22 years | Primary | Science | Moderate |
| **T04** | Male | 3 years | Lower secondary | Information Technology | Low |
| **T05** | Female | 12 years | Upper secondary | English | High |
| **T06** | Female | 18 years | Primary | Mathematics | Moderate |
| **T07** | Male | 6 years | Lower secondary | Integrated Practice | Low |
| **T08** | Female | 25 years | Upper secondary | Physics | Moderate |
| **T09** | Male | 10 years | Primary | Chinese | High |
| **T10** | Female | 5 years | Lower secondary | Information Technology | Low |

Table S10. Open coding

| **ID** | **Original excerpt** | **Interviewee(s)** | **Initial concept** |
| --- | --- | --- | --- |
| **A01** | "The district requires us to use an AI lesson-preparation platform this semester, but I am not even familiar with the basic operations. It takes me a long time every time I log in, and I feel that I have already fallen behind technically." | T01, T02 | Technical operation barriers |
| **A02** | "I recently saw AI systems that can automatically grade math problem-solving questions. It made me wonder whether teachers will no longer even need to look at homework in the future. Then what is our value?" | T05, T06 | Questioning of professional value |
| **A03** | "I am not opposed to using AI, but the fact that it collects student data makes me very uneasy. What if the data are leaked? The school has not given any clear guidance either." | T08, T10 | Data privacy concerns |
| **A04** | "I studied computer science, so using these tools is not difficult for me. The difficult part is how to integrate them into real classroom teaching. Students often respond differently from what I expected." | T07, T09 | Difficulty with classroom adaptation |
| **A05** | "School training only goes through the functions once, with no follow-up support. I am afraid of making mistakes when figuring things out on my own, and colleagues rarely talk about it with each other." | T10, T04 | Sense of isolation in training |
| **A06** | "The equipment in the smart classroom often lags. I carefully prepared an AI interactive activity, but it failed at the critical moment. The students laughed, and I felt very embarrassed." | T09, T06 | Technology reliability anxiety |
| **A07** | "At leadership meetings, AI is emphasized as the future trend, but performance evaluation still depends on test scores and advancement rates. So which direction are we actually supposed to work toward?" | T08, T08 | Misalignment in the evaluation system |
| **A08** | "At my age, having to relearn so many new things is exhausting. It is not that I do not want to learn; I just feel I can no longer keep up." | T09, T01 | Age-related fatigue |
| **A09** | "I teach two classes and also have administrative duties, so I simply do not have time to learn AI tools systematically. When the pressure is high, I do not even feel like trying." | T05, T04 | Time and resource constraints |
| **A10** | "Once I tried using an AI discussion tool, and the classroom response was quite positive. The grade-level coordinator even praised me at a meeting, and after that I felt much more motivated to try other functions." | T02, T05 | Positive feedback incentives |
| **A11** | "Our Chinese language group now sets aside half an hour every week to share small cases of effective AI use. Although the time is short, it makes us feel much more reassured." | T03, T07 | Peer mutual-support |
| **A12** | "During the summer, I attended an AI teaching workshop. It was not about technology itself, but about how to design integrated lesson cases. After I came back, my fear of being 'replaced' was greatly reduced." | T09, T01 | In-depth training alleviates anxiety |
| **A13** | "The issue now is not whether we can use the technology, but how to evaluate teaching effectiveness after using it. The school has no standards, so we do not dare to use it boldly." | T07, T08 | Lack of effectiveness evaluation |
| **A14** | "I think AI should serve as a support tool rather than a dominant force. But current messaging often makes people feel that teachers will be marginalized in the future." | T04, T10 | Concerns about technological dominance |
| **A15** | "The AI scoring system for spoken English is sometimes quite inaccurate. Students do not accept the results, and I have to spend extra time calming them down and explaining things, which actually increases my workload." | T10, T06 | Burden of technological errors |
| **A16** | "I hope the school can establish an AI teaching support group so that we can ask questions whenever problems arise, instead of having to search for everything on our own." | T01, T03 | Need for immediate support |
| **A17** | "Policies change too quickly. Last year we were asked to use one platform, and this year it has already been replaced by another. It feels like we are constantly trying to catch up, and it is exhausting." | T07, T06 | Policy-iteration fatigue |
| **A18** | "Younger teachers adapt quickly, but older teachers worry about holding everyone back. Sometimes we do not dare to use AI in open classes because we are afraid of embarrassment." | T01, T02 | Face-related concerns |
| **A19** | "If AI could truly help me save lesson-preparation time, I would be willing to learn it. But many tools currently just add more steps rather than reducing the burden." | T02, T06 | Expectation of tool practicality |
| **A20** | "I explore AI tools together with my students, and they teach me some novel functions. This shared learning process actually brings us closer." | T02, T04 | Teacher-student co-learning experience |

**Reference**

Beaton, D. E., Bombardier, C., Guillemin, F., & Ferraz, M. B. (2000). Guidelines for the process of cross-cultural adaptation of self-report measures. Spine, 25(24), 3186–3191.

Brislin, R. W. (1970). Back-translation for cross-cultural research. Journal of cross-cultural psychology, 1(3), 185–216.

Cohen, S. (1988). Perceived stress in a probability sample of the United States.

Cohen, S., Kamarck, T., & Mermelstein, R. (1983). A global measure of perceived stress. Journal of health and Social Behavior, 385–396.

Ehrhart, M. G., Aarons, G. A., & Farahnak, L. R. (2014). Assessing the organizational context for EBP implementation: the development and validity testing of the Implementation Climate Scale (ICS). Implementation Science, 9(1), 157.

Podsakoff, P. M., MacKenzie, S. B., Lee, J.-Y., & Podsakoff, N. P. (2003). Common method biases in behavioral research: a critical review of the literature and recommended remedies. Journal of Applied psychology, 88(5), 879.

Schulz, U., & Schwarzer, R. (2003). Social support in coping with illness: the Berlin Social Support Scales (BSSS). Diagnostica, 49(2), 73–82.

Taylor, J. M. (2015). Psychometric analysis of the ten-item perceived stress scale. Psychological assessment, 27(1), 90.

Terzi, R. (2020). An Adaptation of Artificial Intelligence Anxiety Scale into Turkish: Reliability and Validity Study. International Online Journal of Education and Teaching, 7(4), 1501–1515.

Wang, Y.-Y., & Wang, Y.-S. (2022). Development and validation of an artificial intelligence anxiety scale: An initial application in predicting motivated learning behavior. Interactive Learning Environments, 30(4), 619–634.
